# Prediction of plant-level tomato biomass and yield using machine learning with unmanned aerial vehicle imagery

**DOI:** 10.1186/s13007-021-00761-2

**Published:** 2021-07-15

**Authors:** Kenichi Tatsumi, Noa Igarashi, Xiao Mengxue

**Affiliations:** 1 Department of Environmental and Agricultural Engineering, Tokyo University of Agriculture and Technology, 3-5-8 Saiwai-cho, Fuchu, Tokyo 183-8509 Japan; 2 Faculty of Agriculture Tokyo University of Agriculture and Technology, 3-5-8 Saiwai-cho, Fuchu, Tokyo 183-8509 Japan; 3 Graduate School of Agriculture, Tokyo University of Agriculture and Technology, 3-5-8 Saiwai-cho, Fuchu, Tokyo 183-8509 Japan

**Keywords:** Tomato yield prediction, Gray-level co-occurrence matrix, Plant-level, Machine learning, Unmanned aerial vehicle

## Abstract

**Background:**

The objective of this study is twofold. First, ascertain the important variables that predict tomato yields from plant height (PH) and vegetation index (VI) maps. The maps were derived from images taken by unmanned aerial vehicles (UAVs). Second, examine the accuracy of predictions of tomato fresh shoot masses (SM), fruit weights (FW), and the number of fruits (FN) from multiple machine learning algorithms using selected variable sets. To realize our objective, ultra-high-resolution RGB and multispectral images were collected by a UAV on ten days in 2020’s tomato growing season. From these images, 756 total variables, including first- (e.g., average, standard deviation, skewness, range, and maximum) and second-order (e.g., gray-level co-occurrence matrix features and growth rates of PH and VIs) statistics for each plant, were extracted. Several selection algorithms (i.e., Boruta, DALEX, genetic algorithm, least absolute shrinkage and selection operator, and recursive feature elimination) were used to select the variable sets useful for predicting SM, FW, and FN. Random forests, ridge regressions, and support vector machines were used to predict the yield using the top five selected variable sets.

**Results:**

First-order statistics of PH and VIs collected during the early to mid-fruit formation periods, about one month prior to harvest, were important variables for predicting SM. Similar to the case for SM, variables collected approximately one month prior to harvest were important for predicting FW and FN. Furthermore, variables related to PH were unimportant for prediction. Compared with predictions obtained using only first-order statistics, those obtained using the second-order statistics of VIs were more accurate for FW and FN. The prediction accuracy of SM, FW, and FN by models constructed from all variables (rRMSE = 8.8–28.1%) was better than that from first-order statistics (rRMSE = 10.0–50.1%).

**Conclusions:**

In addition to basic statistics (e.g., average and standard deviation), we derived second-order statistics of PH and VIs at the plant level using the ultra-high resolution UAV images. Our findings indicated that our variable selection method reduced the number variables needed for tomato yield prediction, improving the efficiency of phenotypic data collection and assisting with the selection of high-yield lines within breeding programs.

**Supplementary Information:**

The online version contains supplementary material available at 10.1186/s13007-021-00761-2.

## Background

Tomato (*Solanum lycopersicum* L.) is one of the most widely and globally grown vegetables in the world and plays an important role in human health maintenance [1]. In 2018, the annual production of fresh tomatoes was about 180 million tons globally [2]. Approximately a quarter of those were cultivated for processing and were consumed as pastes, ketchup, salsa, and juice [3, 4]. The main production countries are China, India, Pakistan, Turkey, and the U.S., which account for approximately 60% of the world tomato production. Tomato production and harvested area are increasing every year [2]. In terms of health, tomato is a source of vitamin C, potassium, folate, and vitamin K, which have been linked to many health benefits, such as antioxidant protection against cancer, strengthening the heart, and constipation prevention [5].

Over the past few years, unmanned aerial vehicles (UAVs) have been receiving much attention as ways to measure secondary traits, such as plant height (PH) and spectral reflectance, in a wide area because of the UAV advantages: ease of operation, highly flexible and timely control, super-high spatial resolution, and quick retrieval of wide-area field information owing to reduced planning time [6, 7]. A UAV can be equipped with a wide range of sensors useful in agricultural applications, such as RGB [8] and multispectral cameras [9]. In addition, UAVs have attracted much attention in the field of agricultural remote sensing due to the development of low-cost UAVs and imaging sensors. In particular, UAVs provide an entire new perspective to the agricultural landscape by collecting remote sensing data at very low altitudes. Regarding tomato, Senthilnath et al. [10] used a UAV to acquire RGB imagery of a tomato field and to classify tomato and non-tomato plants. However, they found that many fruits were pretermitted because they were visually obscured by leaves and stems. Johansen et al. [11] used a time series of RGB and multispectral datasets to delineate tomato plants using an automated object-based image analysis and to assess phenotypic traits of tomatoes including plant area, growth rates, condition, and plant projective cover. Furthermore, they used the mapped traits to identify tomato plant accessions that performed the best in terms of yield. Johansen et al. [12] researched the predictability of fresh shoot mass (SM), number of fruits (FN), and yield mass at harvest using UAV-based imagery and indicated that plant area, border length, width, and length of plant had the highest importance in the random forest approach to modeling of biomass and yield. Candiago et al. [13] examined the vegetation vigor of vineyards and tomatoes using three different vegetation indices (VIs) based on orthoimages and demonstrated the great potential of high-resolution UAV data. Enciso et al. [14] indicated that canopy cover estimated using a UAV was correlated with measured leaf area index. In other crops, UAV imagery for plant phenotyping has been applied for plant height assessment [15–18], crop growth and biomass, and yield [19–23]. In addition, machine learning (ML) approach with UAV imagery has been used to estimate biomass of crops including wheat [24], rice [25, 26], maize [22], and barley [27]. Except for studies by Moeckel et al. [28] and Johansen et al. [11, 12], we did not identify any studies that used UAV-based time series to predict tomato plant biomass and yield at harvest at the plant level.

The UAV-based studies on yield prediction with remotely sensed phenotypic traits during the growing period used a variety of artificial intelligence approaches, such as ML techniques, and obtained useful findings [29]. In contrast, collecting the required datasets of multitemporal traits needed for large-scale application of ML approaches remains time-consuming and computationally expensive. Currently, if a few principal UAV-derived phenotypic traits and growth stages for crop yield are usable, the data collection and processing effort can be efficient. Furthermore, to reduce computational complexity, improve efficient analysis of data and data understanding, determine essential phenotypic traits or growth stages, variable selection methods involve evaluating important phenotypic traits on yield.

Therefore, although a relatively high number of investigations of optimal variable selection of UAV-derived phenotypic traits and ML for prediction of grain yield have been conducted, only few studies have addressed the leading variable selection on UAV-derived phenotypic traits for prediction of tomato yield. Furthermore, higher-level feature information can be extracted from the ultra-high spatial resolution UAV-acquired imagery at plant level rather than extracting only basic statistics, such as mean and standard deviation, at the plot level. ML algorithms should provide the best predictive results for tomato yield using the selected principal variables. Accurate prediction of tomato yield using sensor-derived secondary traits, such as PH and spectral reflectance, will improve the accuracy genotype selection, shorten the breeding cycle, and reduce the labors in field phenotyping collection and data preprocessing. The specific aims of this study were as follows: (1) to select optimal feature variables for the yield prediction from the UAV-derived PH and VI maps; (2) to evaluate the predictive power for tomato SM, defined as the aboveground biomass except fruit part, fruit weight (FW), and FN using multiple ML algorithms with the set of the selected variables.

## Materials and methods

### Study site and experimental design

**Fig. 1:**
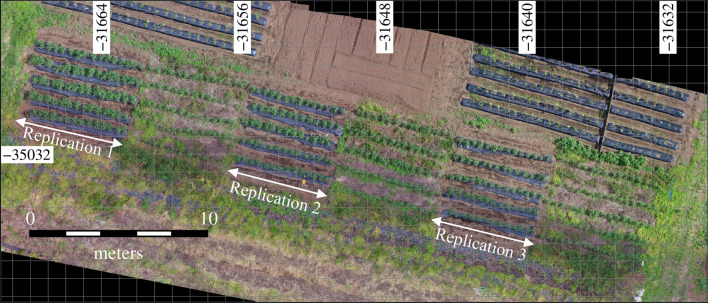
Experimental field used in this study. Field is located in Tokyo, Japan. This orthomosaic image was created using unmanned aerial vehicle images taken on June 18

This study was conducted in an experimental research field at the Field Museum Fuchu, Tokyo University of Agriculture and Technology (35.68°N, 139.48°E). The tomato variety was “Natsunoshun,” which is suitable for processing tomato plants in open fields. The field is of predominantly andosol soil type. Tomato was grown during the growing season from May 13 to July 30, 2020 with three replications and a plot size of 5 × 5 m (Fig. 1). The plants were sown at a greenhouse nursery a month before transplanting, and they were transplanted directly into the ground without staking or trellising tomato plants with bamboo poles or wood stakes. They were arranged in five rows of approximately 0.85 m length with 0.40 m spacing between hills, producing a combined total of 210 plants. Pre-planting N, P, and K fertilizer (N:P:K = 10:10:10 kg 10 a^−1^) was supplied in a split application before transplanting. Drip irrigation was applied through tubing into each plot in the amount of 500 ml for 30 min in the morning and evening for one week after transplanting. Following the initial irrigation period, plots were fed only by rainfall. For each plot, plastic agricultural mulch film was used and weed was periodically manually mowed.

### Data acquisition by UAV

**Fig. 2:**
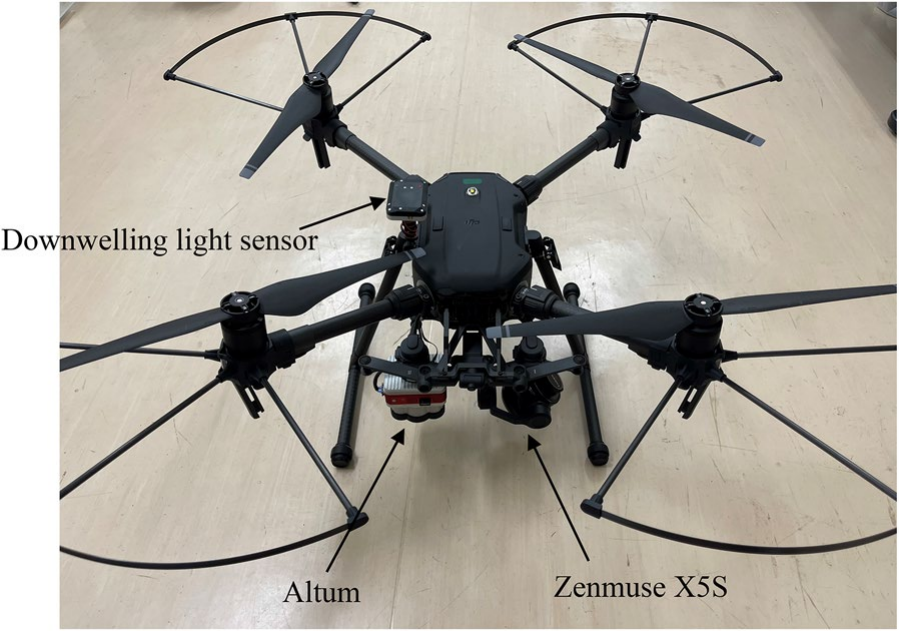
The UAV and sensor system utilized

A gimbal-stabilized Zenmuse X5S camera (DJI Co., Ltd., Shenzhen, China) and multispectral image sensor camera Altum (MicaSense Co., Ltd., SEA, USA) mounted on a DJI Matrice 210 V2 (DJI Co., Ltd., Shenzhen, China) were used to collect the aerial RGB imagery and multispectral images using six bands (blue: 475 nm center, green: 560 nm center, red: 668 nm center, red edge: 717 nm center, near infrared: 842 nm center, and thermal infrared: 8–14 μm). The sensor resolution of each RGB megapixel and individual spectral image (except thermal infrared) was 5280 × 3956 and 2064 × 1554 pixels, respectively. The flight height was 12 m above ground to extract the features of each plant with ultra-high resolution and without being affected by wind caused by the UAV, and the forward and side overlaps were set as 90% and 70%, respectively. The RGB imagery and multispectral reflectance image data were acquired on May 24, May 30, June 5, June 11, June 18, June 26, July 2, July 12, July 16, and July 24. Both types of image data were obtained between 10:00 and 11:00 local time. Aerial multispectral images were radiometrically calibrated with a MicaSense’s Calibrated Reflectance Panel and MicaSense downwelling light sensor mounted on top of the UAV facing up towards the sky (Fig. 2). The reflectance values of the calibrated panel across blue, green, red, near infrared reflectance (nir), and red edge were 0.528, 0.531, 0.531, 0.529, and 0.531, respectively.

Flight parameter settings, including flight path, were designated using the flight planning software Pix4Dcapture (Pix4D S.A., Lausanne, Switzerland), and the ground sampling distance was 0.3 cm/pixel for RGB images and 0.52 cm/pixel for spectral images. Each sensor acquired 80 RGB and 400 spectral images on average per flight. Ground control points, for which we used a black and white cross-centered board, were placed at each of the four corners of the target field. Geometric calibration was conducted during the orthomosaic imagery process in Pix4Dmapper Pro version 4.6.3 (Pix4D S.A., Lausanne, Switzerland) using the ground control points. Digital surface model (DSM), which represents the elevation of plant structures, was created by Pix4Dmapper automatically. The digital terrain model (DTM) which represents the elevation of the soil surface, was estimated by interpolating segmented soil pixels. In this study, the threshold value for segmenting soil and vegetation pixels was set to NDVI = 0.1. For multispectral images, radiometric calibration was performed in Pix4Dmapper during the orthorectification process using the calibration data by panel reflectance values collected during the flight with the downwelling light sensor. Finally, the reflectance map of six-band with GeoTIFF format was obtained automatically using Pix4Dmapper.

### Yield survey

SM, FW, and FN of individual plants in the tomato field were harvested on July 29 and July 30. These yield components were used for variable selection, prediction model training, and prediction accuracy analysis.

### PH and VI calculation

PH maps were calculated by subtracting the DTM from the DSM. The three VIs typically used for measurements of leaf chlorophyll content, plant height, biomass, and crop growth indicators [9, 30–32] were calculated from the multispectral maps: green normalized difference vegetation index (GNDVI) [33], normalized difference vegetation index (NDVI) [34], and weighted difference vegetation index (WDVI) [35]. We selected these three VIs because (1) the use of several types of VIs is not necessarily efficient as time-series analysis takes time, and high correlation coefficients among the VIs causes multicollinearity; (2) NDVI and GNDVI are correlated with leaf chlorophyll content and are widely used for yield predictions [9, 30–32]; 3) WDVI can take into account the soil background influences.

1 $${\text{GNDVI}} = \left( {{\text{NIR}}{-}{\text{Green}}} \right)/\left( {{\text{NIR}} + {\text{Green}}} \right).$$

2 $${\text{NDVI}} = \left( {{\text{NIR}}{-}{\text{Red}}} \right)/\left( {{\text{NIR}} + {\text{Red}}} \right).$$

3 $${\text{WDVI}} = {\text{NIR}}{-}a \times {\text{Red}}{\text{.}}$$

Here, NIR is crop reflectance in the near infrared band, Green is crop reflectance in the green band, Red is crop reflectance in the red band, and *a* is the slope of the soil line.

### Variable extraction from PH and VI maps

In order to extract the variables that may be related to the tomato biomass and yield of each plant, the following preprocessing was performed on the PH and VI maps.

1. Extraction of plant part: Tomato plant parts for each plant were extracted from the pixels with NDVI > 0.5 using the orthomosaic photo image from May 14. Some pixels identified as weeds were manually removed.
2. Centroid determination of each plant: Using the closed vector line of each plant sample obtained in step 1, the centroid of each plant was determined.
3. Region of interest (ROI) extraction of each plant: To calculate the feature variables for each plant, a circle with a radius of 20 cm centered on the centroid of each plant estimated in steps 1 and 2 was extracted as ROI.

In the present study, pixel statistics and dynamic growth rate were extracted as candidates for explanatory variables. In the ROI of each plant, five first-order statistics (average (AVE), standard deviation (SD), skewness (SKEW), range (RANGE), and maximum (MAX)) were extracted as basis statistics from PH and VI maps. Next, thirteen second-order statistics, which only considered the spatial pattern based on gray-level co-occurrence matrix (GLCM) [36], sum average (SA), entropy (Ent), different entropy (DE), sum entropy (SE), variance (Var), difference variance (DV), sum variance (SV), angular second moment (ASM), inverse difference moment (IDM), contrast (Con), correlation (Cor), and information measures of correlation (MOC-1, MOC-2) were derived from PH and VIs maps as GLCM features. Table 1 shows calculation formulas of the 13 extracted feature texture metrics. Next, dynamic average growth rates of PH and VIs were included as second-order statistics in this study and were considered as explanatory variables. In this study, growth rates were calculated for each plot as the change of PH and VIs over two consecutive measurement days divided by the measurement interval. As a result, a total of 756 variables (18 features (first- and second-order statistics) × 10 dates od PH (180 variables); 18 features × 10 days of three VIs (540 variables); 9 dynamic growth rates from PH (9 variables); 9 dynamic growth rates from three VIs (27 variables)) were extracted for variable selection.

**Table 1:** Selected gray-level co-occurrence matrix (GLCM) texture measures and their abbreviations and equations

GLCM feature	Abbreviation	Formula
Sum average	SA	$\mathop \sum \limits_{{k = 0}}^{{2(N - 1)}} k~P_{{x + y}} (k)$
Entropy	Ent	${-}\mathop \sum \limits_{{i = 0}}^{{N{-}1}} \mathop \sum \limits_{{j = 0}}^{{N{-}1}} P_{d} (i,~j)\log ~(P_{d} (i,~j))$
Difference entropy	DE	${-}\mathop \sum \limits_{{k = 0}}^{{N{-}1}} P_{{x{-}y}} (k)\log (P_{{x{-}y}} (k))$
Sum entropy	SE	${-}\mathop \sum \limits_{{k = 0}}^{{2\left( {N{-}1} \right)}} P_{{x + y}} (k)\log (P_{{x + y}} (k))$
Variance	Var	$\mathop \sum \limits_{{i = 0}}^{{N{-}1}} \mathop \sum \limits_{{j = 0}}^{{N{-}1}} (i{-}\mu )^{2} P_{d} (i,j)$
Difference variance	DV	$\mathop \sum \limits_{{k = 0}}^{{N{-}1}} \left( {k~{-}\mathop \sum \limits_{{k = 0}}^{{N{-}1}} k~P_{{x{-}y}} (k)} \right)^{2} P_{{x{-}y}} (k)$
Sum variance	SV	${-}\mathop \sum \limits_{{k = 0}}^{{2(N{-}1)}} \left( {k~{-}\mathop \sum \limits_{{k = 0}}^{{2(N{-}1)}} k~P_{{x + y}} (k)} \right)^{2} P_{{x + y}} (k)$
Angular second moment (uniformity)	ASM	$\mathop \sum \limits_{{i = 0}}^{{N{-}1}} \mathop \sum \limits_{{j = 0}}^{{N{-}1}} P_{d} (i,~j)^{2}$
Inverse difference moment	IDM	$\mathop \sum \limits_{{i = 0}}^{{N{-}1}} \mathop \sum \limits_{{j = 0}}^{{N{-}1}} \frac{1}{{1 + (i~{-}~j)^{2} }}P_{d} (i,~j)$
Contrast	Con	$\mathop \sum \limits_{{k = 0}}^{{N{-}1}} k^{2} P_{x-y} (k)$
Correlation	Cor	$\mathop \sum \limits_{{i = 0}}^{{N{-}1}} \mathop \sum \limits_{{j = 0}}^{{N{-}1}} P_{d} (i,~j)\frac{{(i~{-}~~\mu _{x} )(j~{-}~\mu _{y} )}}{{\sigma _{x} \sigma _{y} }}$
Information measure of correlation-1	MOC-1	$\frac{{HXY~{-}~HXY1}}{{\max (HX,~HY)}}$
Information measure of correlation-2	MOC-2	$[1{-}\exp\{{-}2(HXY2{-}HXY)\}]^{{1/2}}$

*N* is the number of gray levels, $P_d$ is the normalized symmetric GLCM dimension, $P_{d}(i, j)$ is GLCM value on element (*i*, *j*). Other variables were calculated as shown in Additional file 1

### Variable selection for tomato yield prediction

To reduce computational complexity, promote efficient data analysis and data understanding, determine critical phenotypic traits or growth stages, variable selection methods involve evaluating important phenotypic traits on yield. In addition, variable selection is a fundamental step in ML algorithm and regression modeling. In the present study, the two groups of extracted variables, a total of 200 (5 first-order statistics × 10 dates for PH and 3 VI maps) and all 756 available variables, were handled as candidates in variable selection methods to explain tomato SM, FW, and FN. The reason we prepared two groups of variables is to determine if there is a difference in the prediction accuracy of tomato biomass and yield when using only basic statistics and all 756 variables as candidates. After generating first- and second-order statistics and dynamic growth rates from PH and VI maps, normalization was conducted before each variable selection procedure as preprocessing. Next, we applied five effective and powerful variable selection techniques to extract candidate variables. These were Boruta [37], DALEX [38], genetic algorithm (GA) [39], least absolute shrinkage and selection operator (LASSO) [40], and recursive feature elimination (RFE) [41].

Boruta is a non-parametric feature ranking and selection algorithm based on random forest algorithms that can decide if a variable is important and contributes to selection of statistically significant confirmed variables. DALEX is a potent non-parametric tool that explains various attributes such as implemented loss functions about the variables used in a machine learning model. GA is a non-parametric stochastic method for function optimization based on the mechanics of natural genetics and biological evolution. LASSO is a method of automatic parametric variable selection that eventually reduces the coefficients of certain unwanted features to zero due to penalization with L1-norm and minimizes the prediction error. RFE is a non-parametric feature selection that fits a model and removes the weakest feature until the specified number of features is reached. Furthermore, it attempts to eliminate dependencies and collinearity that may exist in the model. All variable selection processes were conducted using R (Version 3.6.3).

In this study, top five variables with the highest importance scores were selected as useful in predicting tomato SM, FW, and FN by five variable selection methods. Each of the variable groups selected from 200 and 756 variables were used as input for random forest (RF), ridge regression (RI), and support vector machine (SVM) in R to predict SM, FW, and FN. RF model is a machine learning method that can be used for a variety of tasks including classification and regression. It consists of a large number of decision trees and combines the predictors of the estimators to produce a more accurate prediction [42]. RI model is a technique for analyzing multiple regression data that suffer from multicollinearity, and it performs L2 regularization [43–45]. SVM model is a supervised machine learning algorithm that is used for classification, regression, and detection of outliers and is capable of addressing the collinearity issue [46]. Hyperparameters for RF, RI, and SVM were optimized using “tuneRF” function in randomForest package, tenfold cross validation, and “tune svm” functions in e1071 package, respectively. To evaluate the model performance, 80% of all observation data were used as training data and the remaining 20% was used for the model evaluation. The prediction performance of each model was evaluated using coefficient of determination (R^2^) and relative root mean square error (rRMSE).

## Results and discussion

### Temporal change of PH and VIs

**Fig. 3:**
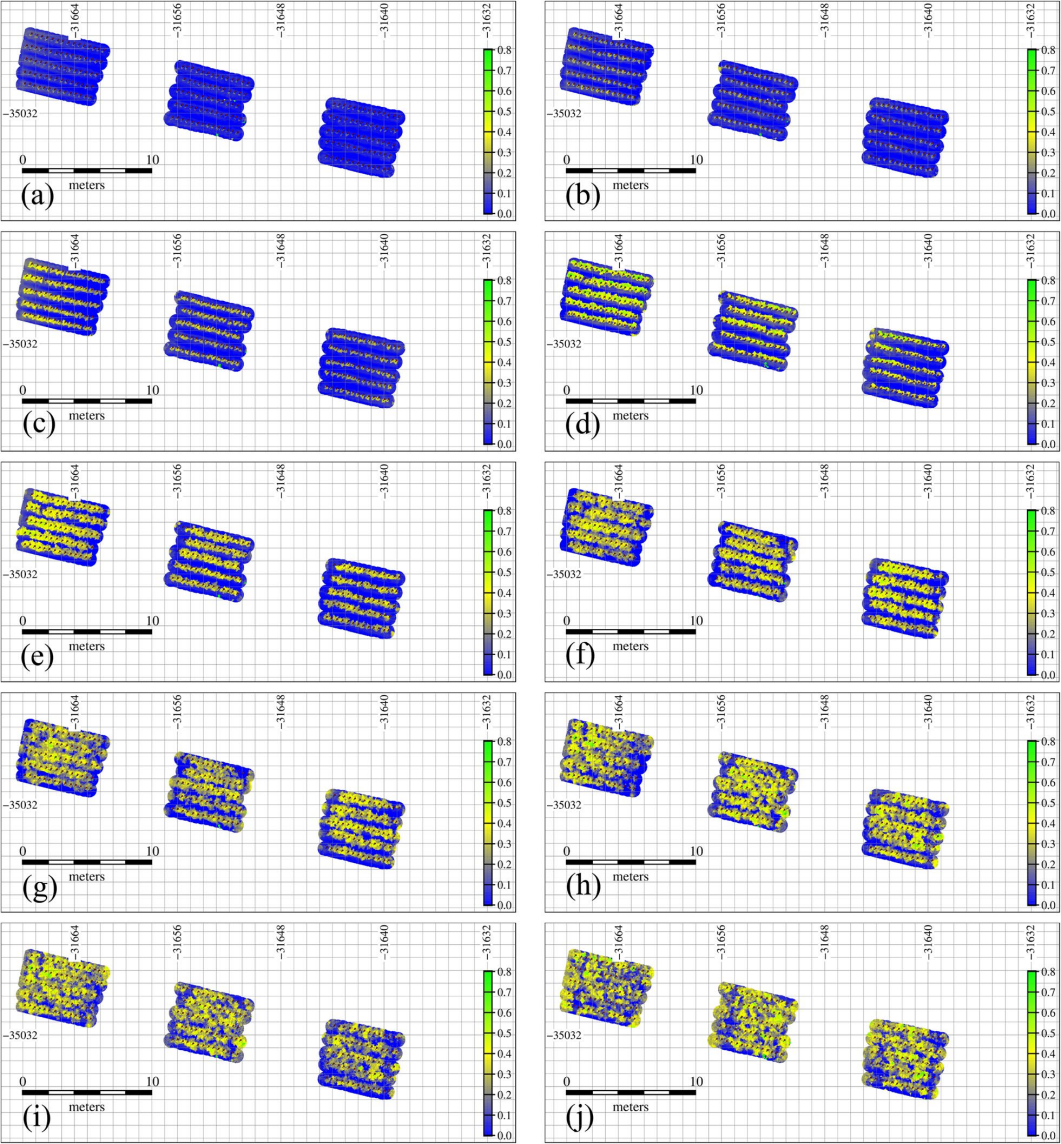
Spatial multitemporal plant height (m). **a** May 24, **b** May 30, **c** June 5, **d** June 11, **e** June 18, **f** June 26, **g** July 2, **h** July 12, **i** July 16, **j** July 24, 2020

**Fig. 4:**
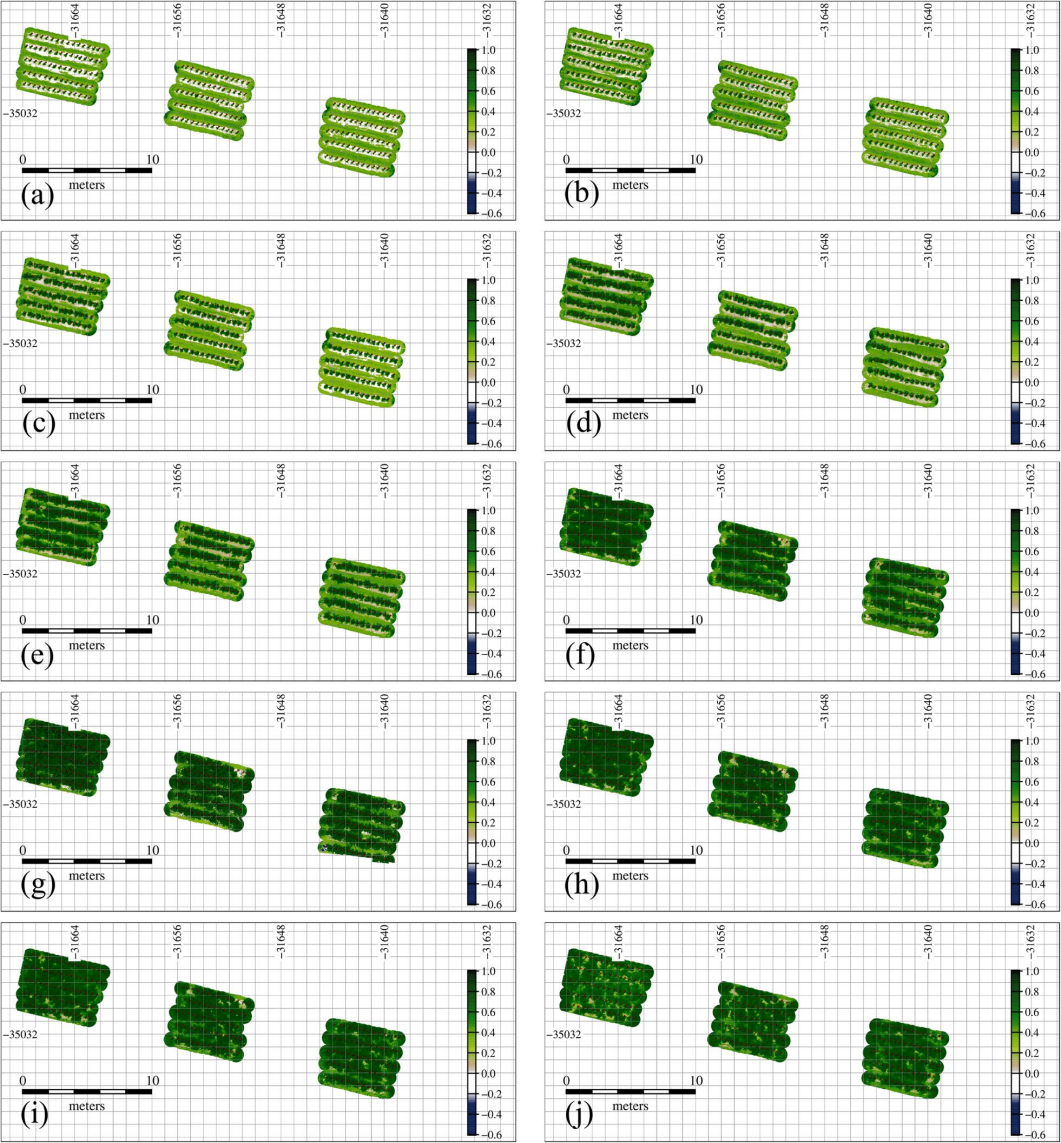
Spatial multitemporal green normalized difference vegetation index (GNDVI) (−). **a** May 24, **b** May 30, **c** June 6, **d** June 11, **e** June 18, **f** June 26, **g** July 2, **h** July 12, **i** July 16, **j** July 24 on 2020

**Fig. 5:**
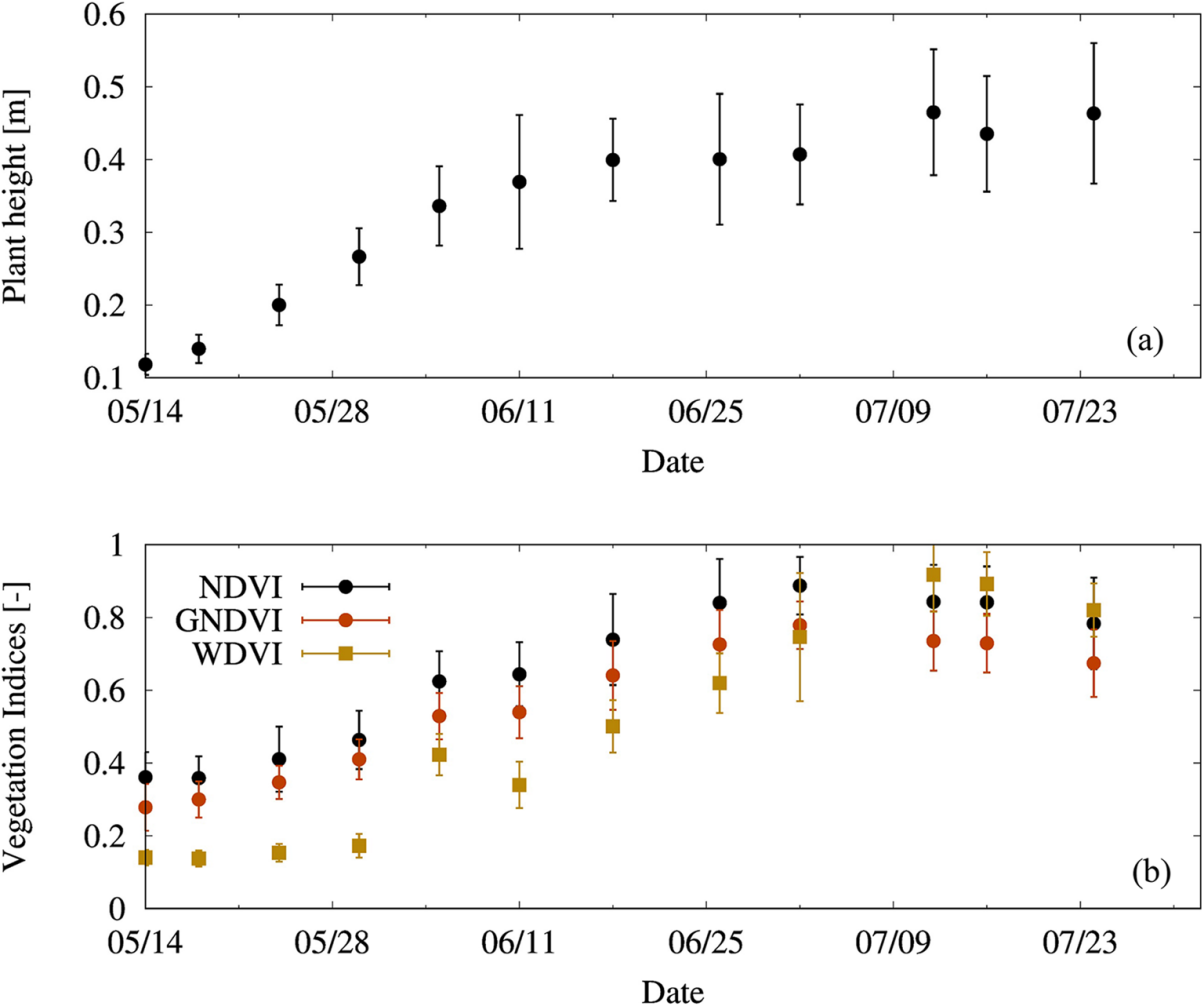
Temporal change of (**a**) plant height and (**b**) three vegetation indices of tomato plants during the growing period. The unmanned aerial vehicle images collected on May 14 and May 18 are not used in the analysis

Multitemporal UAV-derived data allow the quantitative evaluation of tomato growth with PH and VIs using DSM, DTM, and multispectral reflectance images. Figures 3 and 4 show the multitemporal growth change of PH and GNDVI, respectively, during the growing period in the entire field. Additional file 2: Figures S1 and S2 indicate the multitemporal maps of NDVI and WDVI, respectively. Yellower and greener pixels in Figs. 3 and 4, Additional file 2: Figures S1 and S2 mean taller PH and tomato vigor, respectively. PH increased linearly until flowering [around 160 DOY (day of year)]; after that, although the leaves spread horizontally, PH grew at a very small rate and remained almost unchanged during mature fruiting. In contrast, NDVI and GNDVI peaked on 184 DOY, and WDVI peaked on 194 DOY (Fig. 5). The growth trend of PH, which is a sigmoid curve, was also found in other research [47]. In addition, the phenomenon that VIs begin to decline at the end of the growing period is due to the leaf aging and yellowing. In summary, relatively large growth rate of PH and small growth rate of VIs were found until flowering date (mid-June); subsequently, the growth rate of PH slowed down and the growth rates of VIs increased. The ripening stage was characterized by a decrease in the VIs.

### Variable selection

Tables 2, 3, and 4 show the selected top five variables according to averaged importance score estimated by Boruta, DALEX, GA, LASSO, and RFE for SM, FW, and FN, respectively. As for SM, first- and second-order statistics related to PH and VIs during mid-fruit formation stage (from late-June to mid-July) were selected by all variable selection methods. AVE and MAX of PH and RANGE of GNDVI selected from extracted first-order statistics, and SV and DV of NDVI selected from all statistics were ranked in top five by all variable selection methods (Table 2). Moreover, other second-order statistics features such as MOC-1, DV, SE, Entr, IDM, MOC-2, and Con were also ranked as selected variables. Selected first- and second-order statistics of VIs and PH from extracted basic and all variables describe homogeneity and heterogeneity in the entire field. Although basic statistics and GLCM features of PH and VIs are important for SM estimation, all variable selection methods selected more PH-related variables after flowering compared with results of FW and FN. Although these results may vary depending on the employed explanatory variables and the variable selection method, in this study, the importance of PH in SM estimation was confirmed. Prediction of SM is important for estimating the assimilation potential by leaf photosynthesis. Therefore, the relationship of PH and SM has been an interesting issue for researchers and breeders. It was potentially shown that the variables of PH and VIs in the later growth period were significant for the final SM estimation.

**Table 2:** Top five variables selected from first-order statistics and all variables by Boruta, DALEX, genetic algorithm (GA), least absolute shrinkage and selection operator (LASSO), and recursive feature elimination (RFE) for shoot mass (SM)

Rank	From first-order statistics			From first- and second-order statistics		
	Map	Variable	Date	Map	Variable	Date
Boruta						
1	Plant height	AVE	0626	Plant height	MOC-1	0712
2	GNDVI	RANGE	0716	NDVI	SV	0712
3	Plant height	MAX	0720	NDVI	DV	0712
4	GNDVI	AVE	0712	Plant height	AVE	0702
5	NDVI	SD	0712	GNDVI	DV	0724
DALEX						
1	Plant height	AVE	0626	NDVI	SV	0712
2	Plant height	MAX	0702	Plant height	SE	0712
3	Plant height	AVE	0702	NDVI	SE	0712
4	GNDVI	MAX	0530	NDVI	DV	0712
5	GNDVI	RANGE	0530	Plant height	Ent	0626
GA						
1	Plant height	RANGE	0618	Plant height	RANGE	0712
2	Plant height	AVE	0626	NDVI	SV	0712
3	GNDVI	RANGE	0716	NDVI	IDM	0702
4	WDVI	MAX	0724	NDVI	DV	0712
5	NDVI	SD	0712	GNDVI	MOC-2	0724
LASSO						
1	Plant height	AVE	0626	Plant height	AVE	0626
2	GNDVI	RANGE	0716	GNDVI	DV	0724
3	NDVI	MAX	0716	NDVI	SV	0716
4	Plant height	MAX	0702	GNDVI	Con	0618
5	Plant height	SKEW	0605	Plant height	MAX	0702
RFE						
1	Plant height	AVE	0626	NDVI	SV	0712
2	NDVI	MAX	0716	Plant height	MOC-1	0712
3	Plant height	MAX	0702	NDVI	DV	0712
4	Plant height	AVE	0702	NDVI	MAX	0716
5	NDVI	SD	0712	GNDVI	DV	0724

**Table 3:** Top five variables selected from first-order statistics and all variables by Boruta, DALEX, genetic algorithm (GA), least absolute shrinkage and selection operator (LASSO), and recursive feature elimination (RFE) for fruit weight (FW)

Rank	From first-order statistics			From first- and second-order statistics		
	Map	Variable	Date	Map	Variable	Date
Boruta						
1	WDVI	RANGE	0618	WDVI	RANGE	0618
2	NDVI	AVE	0618	NDVI	AVE	0618
3	WDVI	AVE	0618	WDVI	AVE	0618
4	NDVI	AVE	0626	WDVI	SA	0618
5	GNDVI	AVE	0626	NDVI	AVE	0626
DALEX						
1	WDVI	AVE	0618	WDVI	SA	0618
2	NDVI	AVE	0724	NDVI	AVE	0626
3	WDVI	RANGE	0618	NDVI	AVE	0618
4	Plant height	RANGE	0618	WDVI	RANGE	0618
5	NDVI	AVE	0618	GNDVI	IDM	0712
GA						
1	WDVI	RANGE	0618	NDVI	IDM	0716
2	NDVI	MAX	0606	WDVI	RANGE	0618
3	NDVI	AVE	0618	GNDVI	SE	0724
4	NDVI	SD	0716	Plant height	Growth Rate	0530–0605
5	NDVI	SD	0524	WDVI	MAX	0606
LASSO						
1	NDVI	AVE	0618	GNDVI	Con	0618
2	Plant height	MAX	0724	Plant height	MAX	0724
3	NDVI	RANGE	0724	WDVI	SA	0626
4	NDVI	RANGE	0524	NDVI	AVE	0626
5	Plant height	SKEW	0712	NDVI	Cor	0712
RFE	RFE					
1	NDVI	AVE	0618	NDVI	AVE	0618
2	WDVI	RANGE	0618	WDVI	RANGE	0618
3	WDVI	AVE	0618	WDVI	AVE	0618
4	–	–	–	NDVI	AVE	0626
5	–	–	–	WDVI	SA	0618

**Table 4:** Top five variables selected from first-order statistics and all variables by Boruta, DALEX, genetic algorithm (GA), least absolute shrinkage and selection operator (LASSO), and recursive feature elimination (RFE) for number of fruit (FN)

Rank	From first-order statistics			From first- and second-order statistics		
	Map	Variable	Date	Map	Variable	Date
Boruta						
1	NDVI	AVE	0626	WDVI	RANGE	0618
2	GNDVI	AVE	0626	NDVI	AVE	0618
3	NDVI	MAX	0618	WDVI	AVE	0618
4	GNDVI	MAX	0611	WDVI	SA	0618
5	GNDVI	SD	0626	NDVI	AVE	0626
DALEX						
1	NDVI	RANGE	0606	WDVI	IDM	0618
2	NDVI	AVE	0626	NDVI	RANGE	0626
3	NDVI	AVE	0524	NDVI	AVE	0618
4	NDVI	MAX	0618	WDVI	AVE	0618
5	NDVI	MAX	0618	GNDVI	SA	0712
GA						
1	WDVI	SD	0712	NDVI	IDM	0716
2	NDVI	SD	0524	WDVI	RANGE	0618
3	WDVI	MAX	0606	GNDVI	AVE	0724
4	GNDVI	AVE	0626	Plant height	Growth rate	0530–0605
5	GNDVI	RANGE	0524	WDVI	MAX	0606
LASSO						
1	GNDVI	MAX	0611	GNDVI	Con	0618
2	GNDVI	AVE	0626	Plant height	MAX	0724
3	WDVI	SD	0606	WDVI	SA	0626
4	GNDVI	AVE	0712	NDVI	AVE	0626
5	NDVI	MAX	0606	NDVI	Cor	0712
RFE						
1	GNDVI	AVE	0626	NDVI	AVE	0618
2	NDVI	AVE	0626	WDVI	RANGE	0618
3	NDVI	MAX	0618	WDVI	AVE	0618
4	NDVI	MAX	0606	NDVI	AVE	0626
5	NDVI	SD	0626	WDVI	SA	0618

In contrast, most selected variables by all variable selection models for FW were VI-related variables. RANGE of WDVI on June 18 selected by Boruta, DALEX, GA, and RFE from basic and all variables was ranked in top five variables (Table 3). Moreover, AVE of NDVI was also ranked in top five variables by all variable selection models except for GA from all available variables. Interestingly, the results show that VI variables about one month prior to harvest are critical for estimating FW. The finding that the VIs at the onset of fruiting are determinants of the final FW can be relevant for field management. Similar to FW, FN is an important factor for determining yield. Technology to estimate the FN on each plant, which is not visible in aerial images, can contribute to cultivation management. Although the selected variables by variable selection methods are different, AVE of NDVI or WDVI was selected in top five variables from both basic and all available variables (Table 4). In addition, it can be seen that the first- and second-order statistics of VIs on early to mid-fruit formation period are useful for estimating FN. However, unlike with SM, PH-related variables were found to be unnecessary variables for estimating FN.

### Tomato yield prediction by models using selected variables

**Fig. 6:**
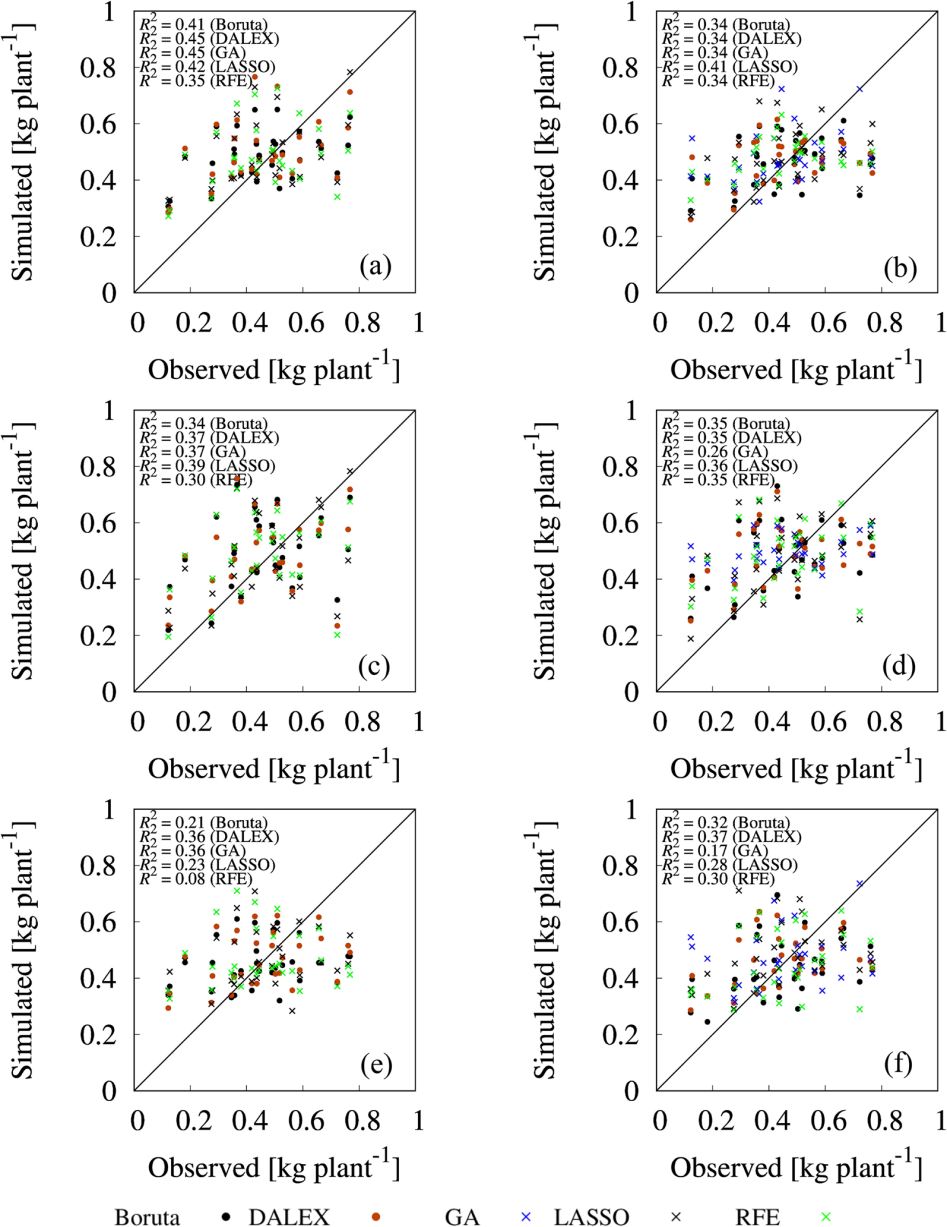
Correlations between observed and simulated plant weight: **a** random forest (RF) with selected variables from first-order statistics. **b** RF with selected variables from first- and second-order statistics. **c** ridge regression (RI) with selected variables from first-order statistics. **d** RI with selected variables from first- and second-order statistics. **e** support vector machine (SVM) with selected variables from first-order statistics. **f** SVM with selected variables from first- and second-order statistics

**Fig. 7:**
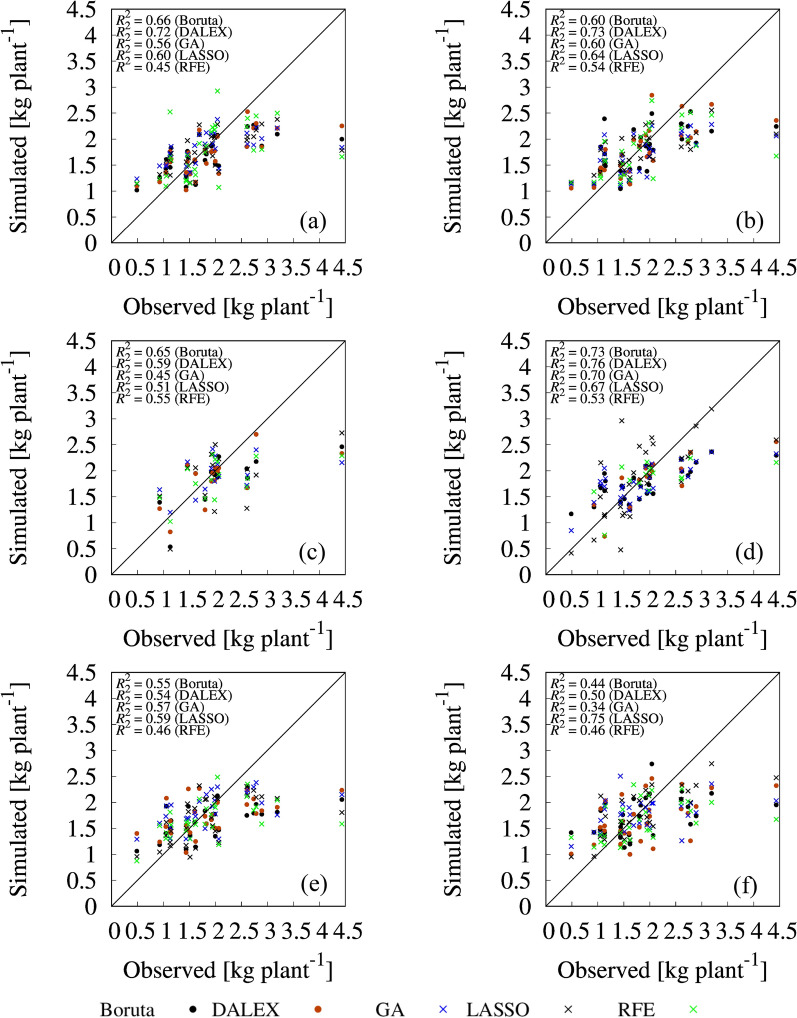
Correlations between observed and simulated fruit weight. **a** Random forest (RF) with selected variables from first-order statistics. **b** RF with selected variables from first- and second-order statistics. **c** Ridge regression (RI) with selected variables from first-order statistics. **d** RI with selected variables from first- and second-order statistics. **e** Support vector machine (SVM) with selected variables from first-order statistics. **f** SVM with selected variables from first- and second-order statistics

**Fig. 8:**
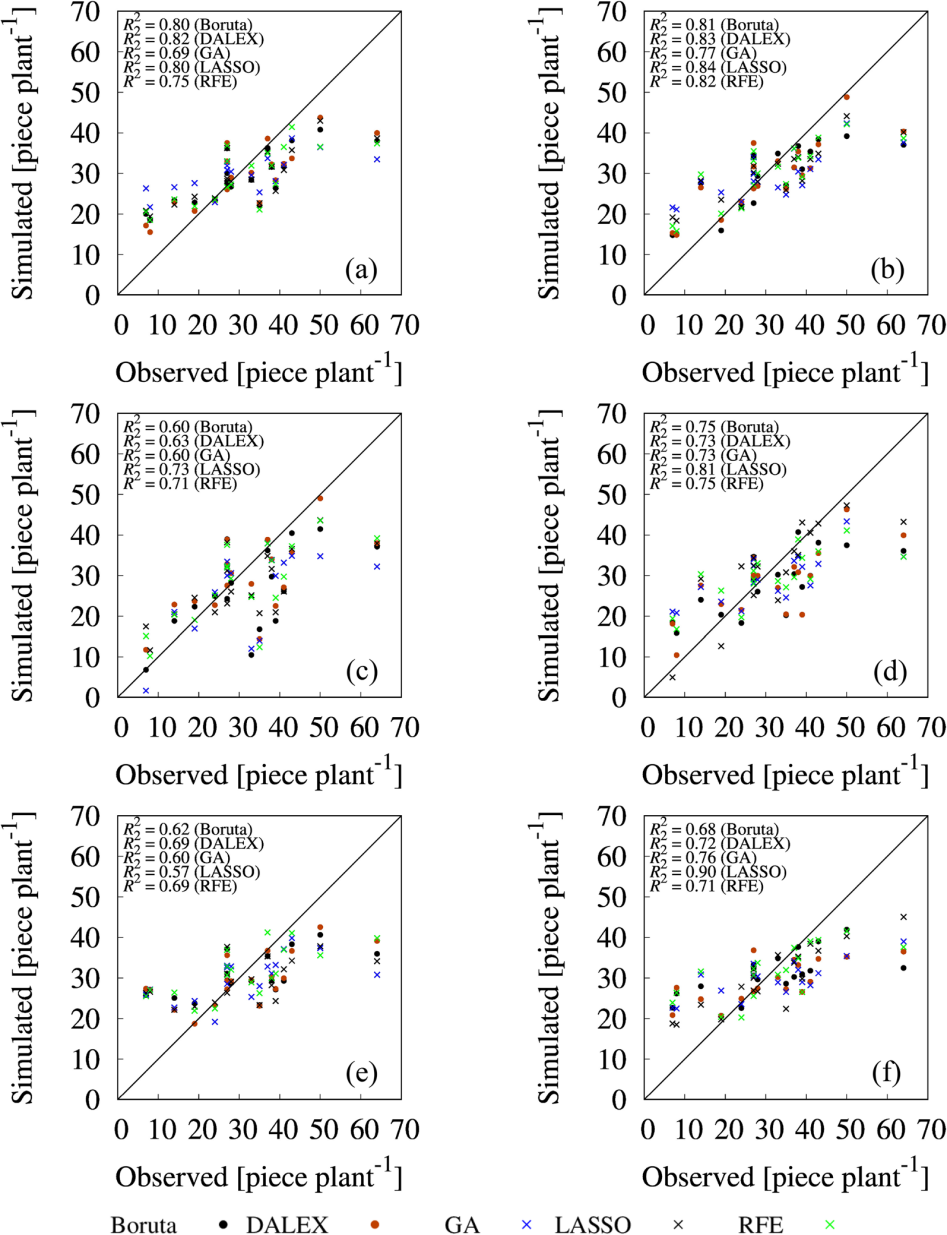
Correlations between observed and simulated number of fruits. **a** Random forest (RF) with selected variables from first-order statistics. **b** RF with selected variables from first- and second-order statistics. **c** Ridge regression (RI) with selected variables from first-order statistics. **d** RI with selected variables from first- and second-order statistics. **e** support vector machine (SVM) with selected variables from first-order statistics. **f** SVM with selected variables from first- and second-order statistics

RF, RI, and SVM were built by using 80% (*n* = 120) of the data for training with a total of five sets of selected important variables. The performance of constructed models was evaluated using the remaining 20% for testing by tenfold cross validation. Figures 6, 7, and 8 show the relationship between observed and simulated SM, FW, and FN using RF, RI, and SVM models with the selected variables set, respectively. Table 5 indicates rRMSE between the observed and simulated values by RF, RI, and SVM models with the five sets of selected variables, reflecting the prediction accuracy of the validated model with test data.

**Table 5:** relative Root mean square error (rRMSE) value of tomato shoot mass (SM), fruit weight (FW), and number of fruits (FN) using random forest (RF), ridge regression (RI), and support vector machine (SVM) models with selected variables set from first-order statistics and all variables

Model	From first-order statistics				
	Boruta	DALEX	GA	LASSO	RFE
SM [kg plant^−1^]					
RF	17.8	22.2	16.9	22.5	22.9
RI	26.4	26.7	24.9	30.6	26.7
SVM	17.6	18.9	16.7	21.4	21.8
FW [kg plant^−1^]					
RF	14.0	13.9	13.2	15.7	24.1
RI	49.6	48.5	48.5	50.1	48.0
SVM	14.3	14.5	14.6	18.7	15.9
FN [piece plant^−1^]					
RF	12.6	14.2	10.0	12.4	14.2
RI	30.4	25.5	30.3	18.1	21.2
SVM	13.1	14.2	13.5	13.0	13.6

Model	From first- and second-order statistics				
	Boruta	DALEX	Genetic	LASSO	RFE
SM [kg plant^−1^]					
RF	18.7	16.5	15.0	13.6	13.4
RI	22.7	20.5	11.0	25.8	17.6
SVM	21.4	18.7	11.2	21.6	20.5
FW [kg plant^−1^]					
RF	18.0	15.6	12.5	13.8	15.6
RI	11.5	20.6	12.8	22.1	16.7
SVM	14.7	15.4	14.6	15.9	14.5
FN [piece plant^−1^]					
RF	14.9	13.5	11.2	11.5	13.5
RI	15.5	17.7	12.7	28.1	13.4
SVM	12.8	10.9	8.8	10.6	14.1

Comparing among the five different variable sets for SM prediction, variable sets from first-order statistics selected by Boruta and DALEX had better goodness of fit with RF model (R^2^ = 0.41 for Boruta; R^2^ = 0.45 for DALEX) (Fig. 6a) than the other combinations of variable selection method and prediction model. In the rRMSE, GA-selected variable set from all variables with RI and SVM had smaller absolute error of a model (rRMSE = 11.0% for RI; rRMSE = 11.2% for SVM). For SM prediction, RF with selected variable sets from first-order basic statistics had better performance in R^2^; whereas second-order statistics decreased the rRMSE value for many combinations of prediction models and variable selection methods (Table 5). For the comparison with R^2^, it was an interesting result that prediction of SM does not require the GLCM texture information, and only the first-order statistics are sufficient to obtain the certain prediction accuracy.

Regarding FW, the following combinations had superior performance among the variable sets and prediction models combinations: Boruta-, DALEX-, and GA-selected variable sets from all variables with RI (R^2^ = 0.73, 0.76, and 0.70, respectively) (Fig. 7d), LASSO-selected variable set from all variables with SVM (R^2^ = 0.75) (Fig. 7f), and RFE-selected variable set from first-order statistics variables with RI (R^2^ = 0.55) (Fig. 7c). In particular, focusing on the RI, the prediction accuracy of the models, except RFE, with the selected variables using all variables was greatly improved compared with that using selected variable set from first-order statistics only. For example, GA-selected variable set from first-order statistics with RI model had lower R^2^ value (0.45), whereas that from all variables had higher R^2^ value (0.70). IDM of NDVI from all variables was ranked as top one variable by GA (Table 3). IDM feature relates inversely to the contrast measure and is a direct measure of the local homogeneity of a digital image. Therefore, this result shows the importance of second-order statistics for predicting FW. For FN prediction, simulated value with selected variable sets from all variables by all prediction models had significantly higher goodness of fit compared with selected variable sets from first-order statistics. In particular, Boruta-, DALEX-, GA-, and RFE-selected important variable sets with RF (Fig. 8b), and LASSO-selected feature variable sets with SVM (Fig. 8f) achieved higher prediction performances compared with the other combinations of selected feature variable sets and prediction models (R^2^ = 0.81 for Boruta; R^2^ = 0.83 for DALEX; R^2^ = 0.82 for RFE; R^2^ = 0.77 for GA; R^2^ = 0.82 for RFE; R^2^ = 0.90 for LASSO).

In the present study, RF with Boruta-selected variable set from first-order statistics, RI with DALEX-selected variable set from all variables, and SVM with LASSO-selected variable set from all variables had best prediction performance R^2^ with the observed SM, FW, and FN, respectively. Although it is difficult to make simple comparisons due to the differences of cultivation environments, varieties, and extracted variables, Li et al. [48] suggested that non-parametric (parametric) prediction model is adopted to match the non-parametric (parametric) variable selection. In this study, there were no clear effect relationships between parametric (non-parametric) variable selection method and parametric (non-parametric) model on tomato yield prediction accuracy. Furthermore, prediction accuracy of FW and FN using the selected variable set from all variables was significantly better compared with that using selected variable set from first-order statistics. Moreover, statistic variables of the VIs about one month before harvest were found to be important in predicting tomato yield.

Narrowing the focus to secondary traits and growth stages that affect tomato yield will contribute to more effective phenotypic data collection. In addition, the super-high-resolution field images obtained from the UAV provided helpful traits, such as temporal change of plant height and vegetation indices including secondary-order statistics of the field. Although we only used these three VIs in this study, we intend to use more VIs, including blue, red edge, and thermal bands, to predict the yield in the near future. Our next goal is to extract the features necessary to build a robust prediction model by testing the proposed variable selection with more data collected in multipoint and multiple years and thus contribute to the efficient selection of high-yield lines in breeding process.

## Conclusion

In this study, we sought to examine the prediction accuracy of SM, FW, and FN using RF, RI, and SVM using variable sets selected by Boruta, DALEX, GA, LASSO, and RFE. PH and VIs (NDVI, GNDVI, and WDVI) from UAV-derived imagery were used for extraction of first-order basic statistics and second-order statistics (GLCM features and dynamic growth rate). First-order statistics of PH and VIs at early to mid-fruit formation period were ranked as important variables for prediction of SM by all feature selection methods. GLCM features of NDVI and WDVI from June 18 were significantly important for prediction of FW. Similar to FW prediction, GLCM features of VIs one month before harvest were significant to predict FN. Furthermore, all prediction models with the selected variable sets from all variables achieved good performance for FW and FN prediction compared with selected variable sets using basic statistics only. In particular, RF with Boruta-selected variable set from the basic statistics, RI with DALEX-selected variable set from all variables, and SVM with LASSO-selected variable set from all variables were best combinations for predicting SM, FW, and FN, respectively. These results indicate that filtering secondary traits and growth stages that contribute to the prediction of tomato yield can contribute to saving of time and labor required for phenotypic data collection and processing. In addition, it is possible to obtain useful features for breeding, other than first-order basic statistics, such as second-order statistics in PH and VIs for each plant, from the ultra-high-resolution image obtained by UAV. Overall, our findings indicate that reduced features needed for tomato yield prediction by variable selection method will help improve the efficiency of phenotypic data collection and assist with the selection of high-yield lines in breeding programs.

In traditional machine learning techniques, it is difficult to apply a trained model to other tomato cultivars or other crop species. In a future study, we will apply transfer learning techniques to extract the phenotype of different domains quantitatively using an expanded UAV dataset, and we will compare the results with those of the process in this study.

## Supplementary Information

**Additional file 1.** Auxiliary formula for calculation of gray-level co-occurrence matrix (GLCM) features.

**Additional file 2: Figure S1.** Spatial multitemporal normalized difference vegetation index (NDVI) (−). **Figure S2.** Spatial multitemporal weighted difference vegetation index (WDVI) (−).

## Data Availability

The all datasets used analyzed in this study are available from the corresponding author on reasonable request.
