# Supplementary material for: Prediction of plant-level tomato biomass and yield using machine learning with unmanned aerial vehicle imagery
Source: Plant Methods. 2021 Jul 15;17:77. doi: 10.1186/s13007-021-00761-2 (PMC8281694; doi:10.1186/s13007-021-00761-2)
Supplement: Supplementary file 1 — Additional file 1. Auxiliary formula for calculation of gray-level co-occurrence matrix (GLCM) features. [file 13007_2021_761_MOESM1_ESM.docx]

**Auxiliary formula for calculation of gray-level co-occurrence matrix (GLCM) features**

(1)

(2)

(3)

(4)

(5)

(6)

(7)

(8)

(9)

(10)

(11)

$$\text{P}_{\text{x}}\text{(}\text{i}\text{)}\text{ }\text{=}\sum_{\text{j}\text{=0}}^{\text{N}\text{–}\text{1}} \text{P}_{\text{d}}\text{(}\text{i}\text{,}\text{ }\text{j}\text{)}$$

$$\text{P}_{\text{y}}\text{(}\text{j}\text{)}\text{ }\text{=}\sum_{\text{i}\text{=0}}^{\text{N}\text{–}\text{1}} \text{P}_{\text{d}}\text{(}\text{i}\text{,}\text{ }\text{j}\text{) }$$

$$\text{P}_{\text{x}\text{+}\text{y}}\left( \text{k} \right)\text{ }\text{=}\sum_{\text{i}\text{=0}}^{\text{N}\text{–}\text{1}} \sum_{\text{j}\text{=0}}^{\text{N}\text{–}\text{1}} \text{P}_{\text{d}}\text{(}\text{i}\text{,}\text{ }\text{j}\text{)}\text{, }\text{k}\text{ }\text{=}\text{ }\text{i}\text{ }\text{+}\text{ }\text{j}\text{ }\text{=}\text{ }\text{0,}\text{ }\text{1}\text{ }\text{,…,}\text{ }\text{2(}\text{N}\text{–}\text{1)}$$

$$\text{P}_{\text{x}\text{–}\text{y}}\left( \text{k} \right)\text{ }\text{=}\sum_{\text{i}\text{=0}}^{\text{N}\text{–}\text{1}} \sum_{\text{j}\text{=0}}^{\text{N}\text{–}\text{1}} \text{P}_{\text{d}}\text{(}\text{i}\text{,}\text{ }\text{j}\text{)}\text{, }\text{k}\text{ }\text{=}\text{ }\text{i}\text{ }\text{–}\text{ }\text{j}\text{ }\text{=}\text{ }\text{0,}\text{ }\text{1}\text{ }\text{,…,}\text{ }\text{N}\text{–}\text{1}$$

$$\text{ }\text{HX}\text{ }\text{= }\text{–}\sum_{\text{i}\text{=0}}^{\text{N}\text{–}\text{1}} \text{P}_{\text{x}}\left( \text{i} \right)\log\text{(}\text{P}_{\text{x}}\text{(}\text{i}\text{))}$$

$$\text{ }\text{HY}\text{ }\text{= }\text{–}\sum_{\text{j}\text{=0}}^{\text{N}\text{–}\text{1}} \text{P}_{\text{y}}\left( \text{j} \right)\log\text{(}\text{P}_{\text{y}}\text{(}\text{j}\text{))}$$

$$\text{ }\text{HXY}\text{ }\text{=}\text{ }\text{–}\text{ }\sum_{\text{i}\text{=0}}^{\text{N}\text{–}\text{1}} \sum_{\text{j}\text{=0}}^{\text{N}\text{–}\text{1}} \text{P}_{\text{d}}\left( \text{i}\text{,}\text{ }\text{j} \right)\text{ }\text{log(}\text{P}_{\text{d}}\left( \text{i}\text{,}\text{ }\text{j} \right)\text{)}$$

$$\text{ }\text{HXY}\text{1=}\text{ }\text{–}\text{ }\sum_{\text{i}\text{=0}}^{\text{N}\text{–}\text{1}} \sum_{\text{j}\text{=0}}^{\text{N}\text{–}\text{1}} \text{P}_{\text{d}}\left( \text{i}\text{,}\text{ }\text{j} \right)\text{ }\text{log(}\text{P}_{\text{x}}\left( \text{i} \right){\text{ }\text{P}}_{\text{y}}\left( \text{j} \right)\text{)}$$

$$\text{ }\text{HXY}\text{2}\text{ }\text{=}\text{ }\text{–}\text{ }\sum_{\text{i}\text{=0}}^{\text{N}\text{–}\text{1}} \sum_{\text{j}\text{=0}}^{\text{N}\text{–}\text{1}} \text{P}_{\text{x}}\left( \text{i} \right){\text{ }\text{P}}_{\text{y}}\left( \text{j} \right)\text{ }\text{log(}\text{P}_{\text{x}}\left( \text{i} \right){\text{ }\text{P}}_{\text{y}}\left( \text{j} \right)\text{)}$$

$${\text{ }\text{μ}}_{\text{x}}= \sum_{\text{i}\text{=0}}^{\text{N}\text{–}\text{1}} \sum_{\text{j}\text{=0}}^{\text{N}\text{–}\text{1}} {\text{i}\text{ }\text{P}}_{\text{d}}\text{(}\text{i}\text{,}\text{ }\text{j}\text{)}$$

$${\text{ }\text{μ}}_{\text{y}}= \sum_{\text{i}\text{=0}}^{\text{N}\text{–}\text{1}} \sum_{\text{j}\text{=0}}^{\text{N}\text{–}\text{1}} {\text{i}\text{ }\text{P}}_{\text{d}}\text{(}\text{i}\text{,}\text{ }\text{j}\text{)}$$

$$\text{σ}_{\text{x}}= \sqrt{\sum_{\text{i}\text{=0}}^{\text{N}\text{–}\text{1}} \sum_{\text{j}\text{=0}}^{\text{N}\text{–}\text{1}} \text{(}\text{i}\text{ }\text{–}\text{ }\text{μ}\text{)}^{\text{2}}\text{P}_{\text{d}}\text{(}\text{i}\text{,}\text{ }\text{j}\text{)}}$$

$$\text{σ}_{\text{y}}= \sqrt{\sum_{\text{i}\text{=0}}^{\text{N}\text{–}\text{1}} \sum_{\text{j}\text{=0}}^{\text{N}\text{–}\text{1}} \text{(}\text{j}\text{ }\text{–}\text{ }\text{μ}\text{)}^{\text{2}}\text{P}_{\text{d}}\text{(}\text{i}\text{,}\text{ }\text{j}\text{)}}$$

(12)

(13)
