# Supplementary material for: Prediction of plant-level tomato biomass and yield using machine learning with unmanned aerial vehicle imagery
Source: Plant Methods. 2021 Jul 15;17:77. doi: 10.1186/s13007-021-00761-2 (PMC8281694; doi:10.1186/s13007-021-00761-2)
Supplement: Supplementary file 2 — Additional file 2: Figure S1. Spatial multitemporal normalized difference vegetation index (NDVI) (−). Figure S2. Spatial multitemporal weighted difference vegetation index (WDVI) (−). [file 13007_2021_761_MOESM2_ESM.docx]

| 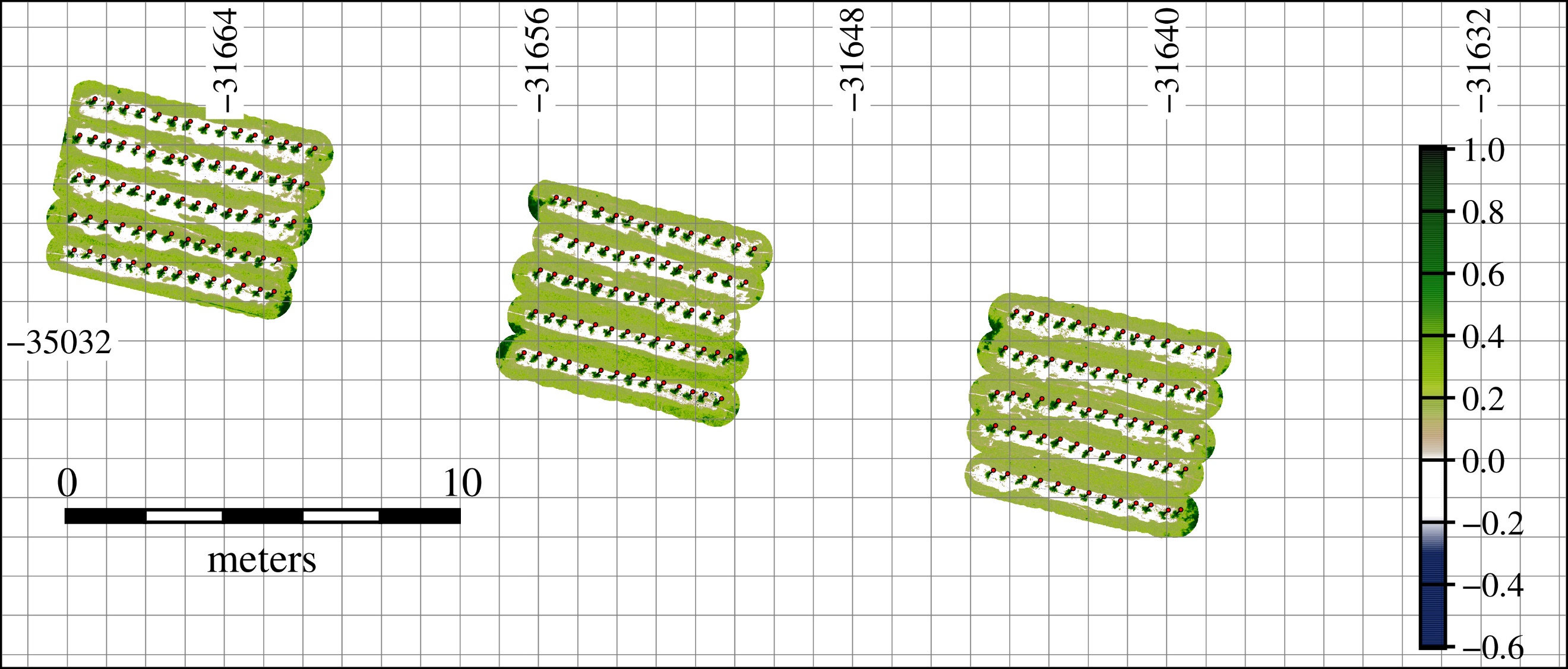  (a) | 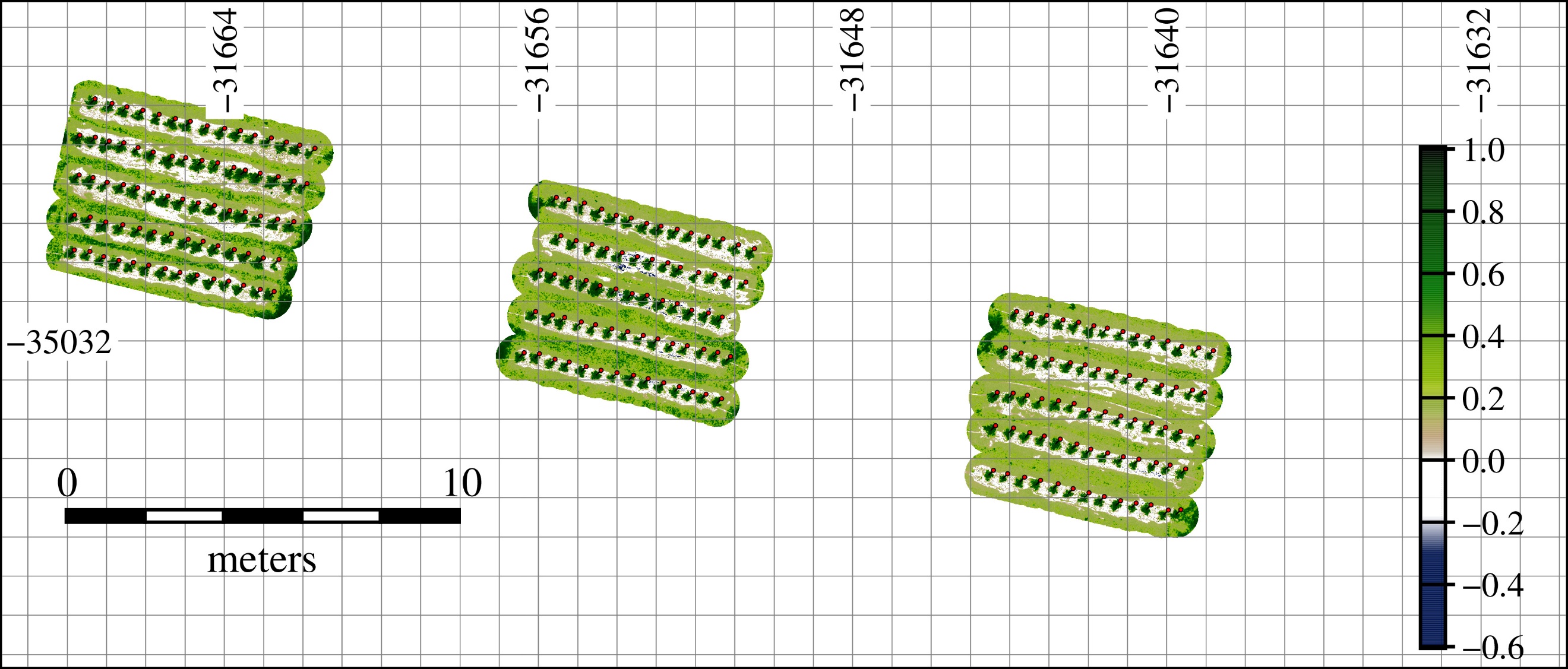  (b) |
| --- | --- |
| 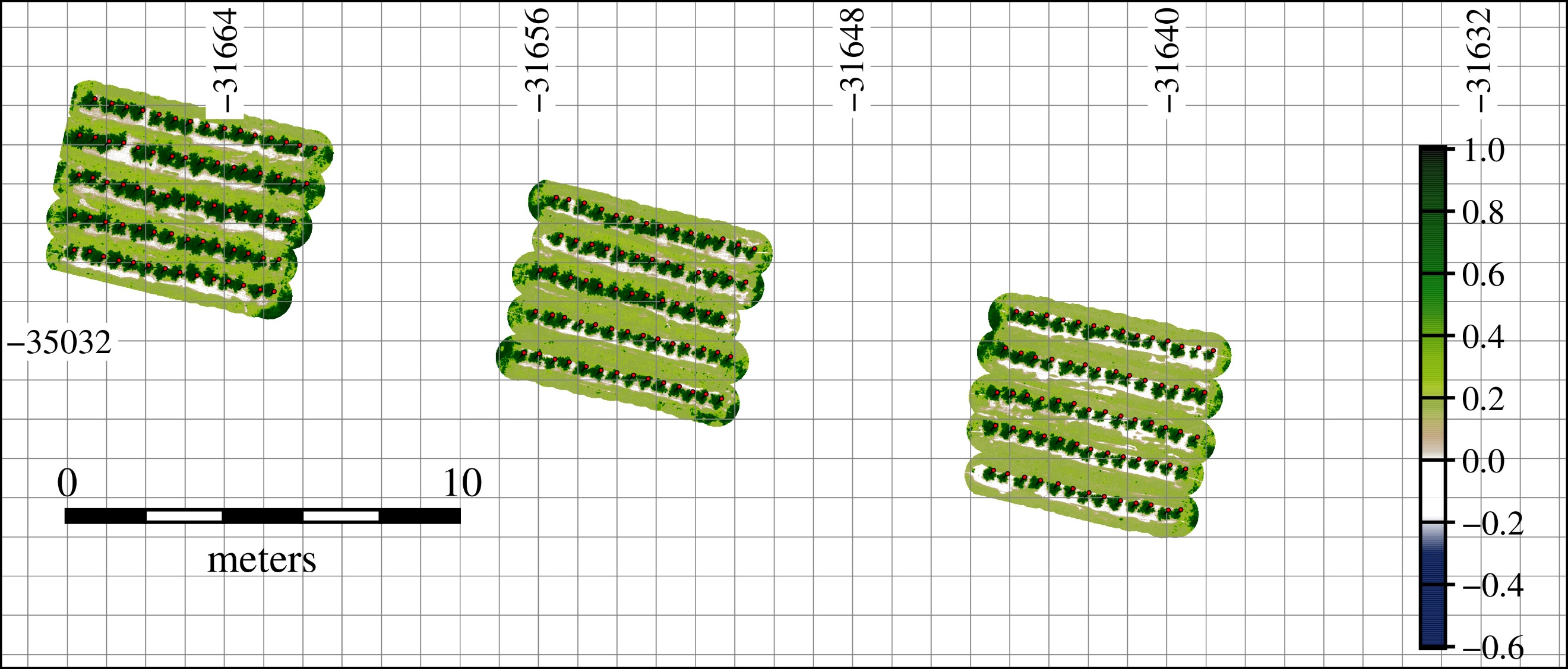  (c) | 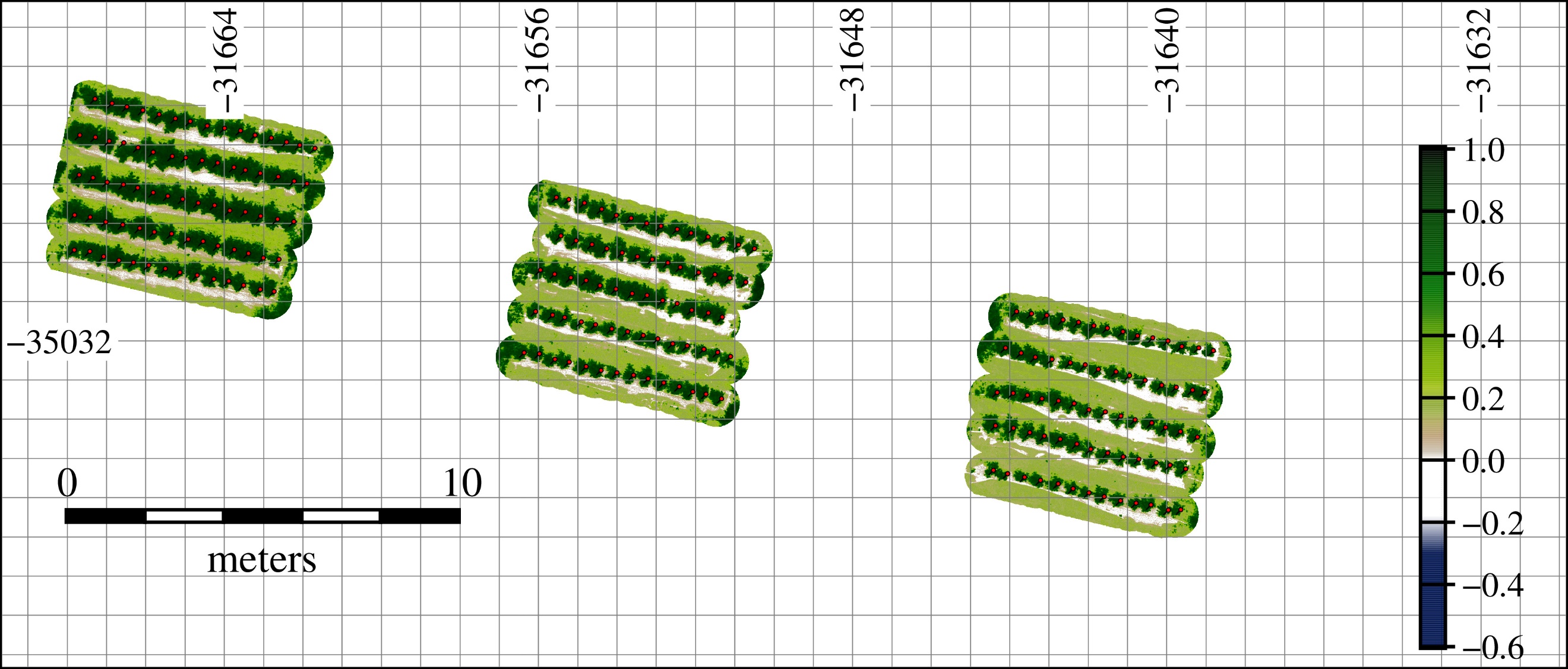  (d) |
| 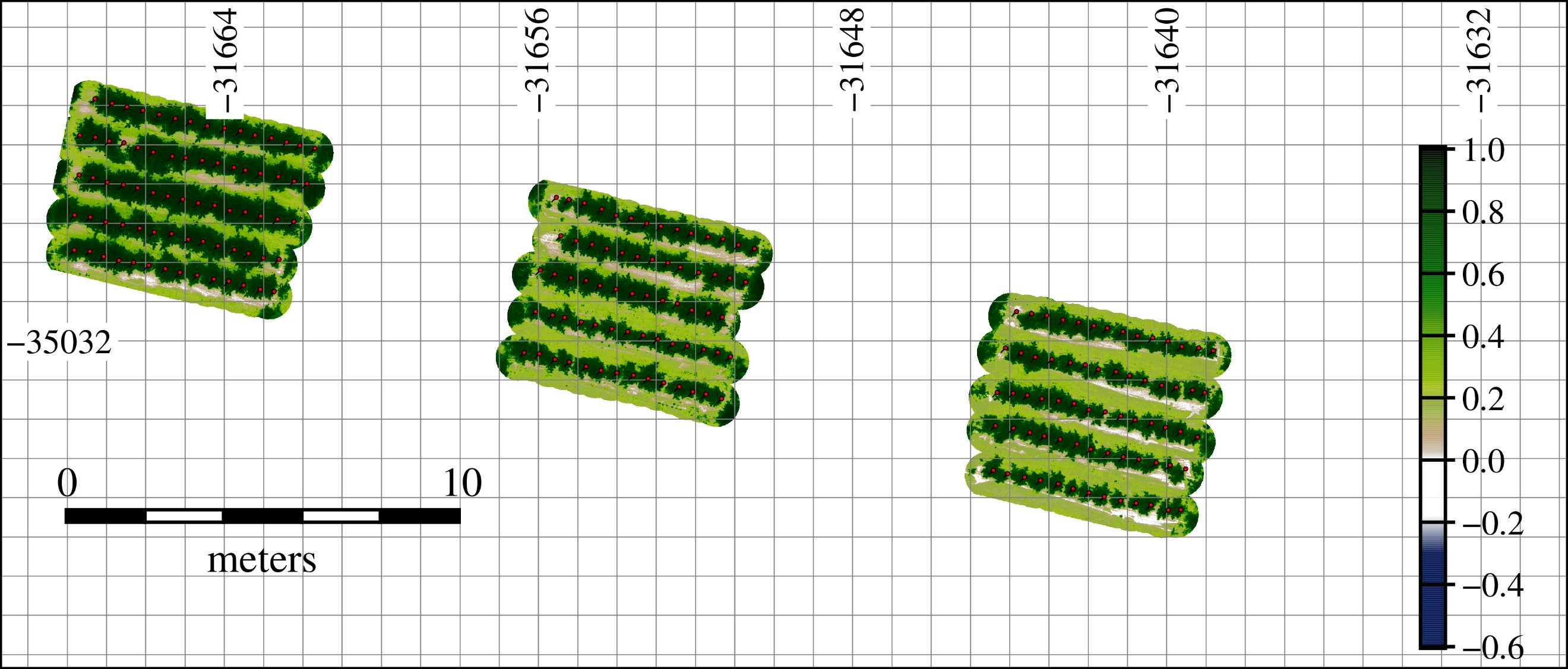  (e) | 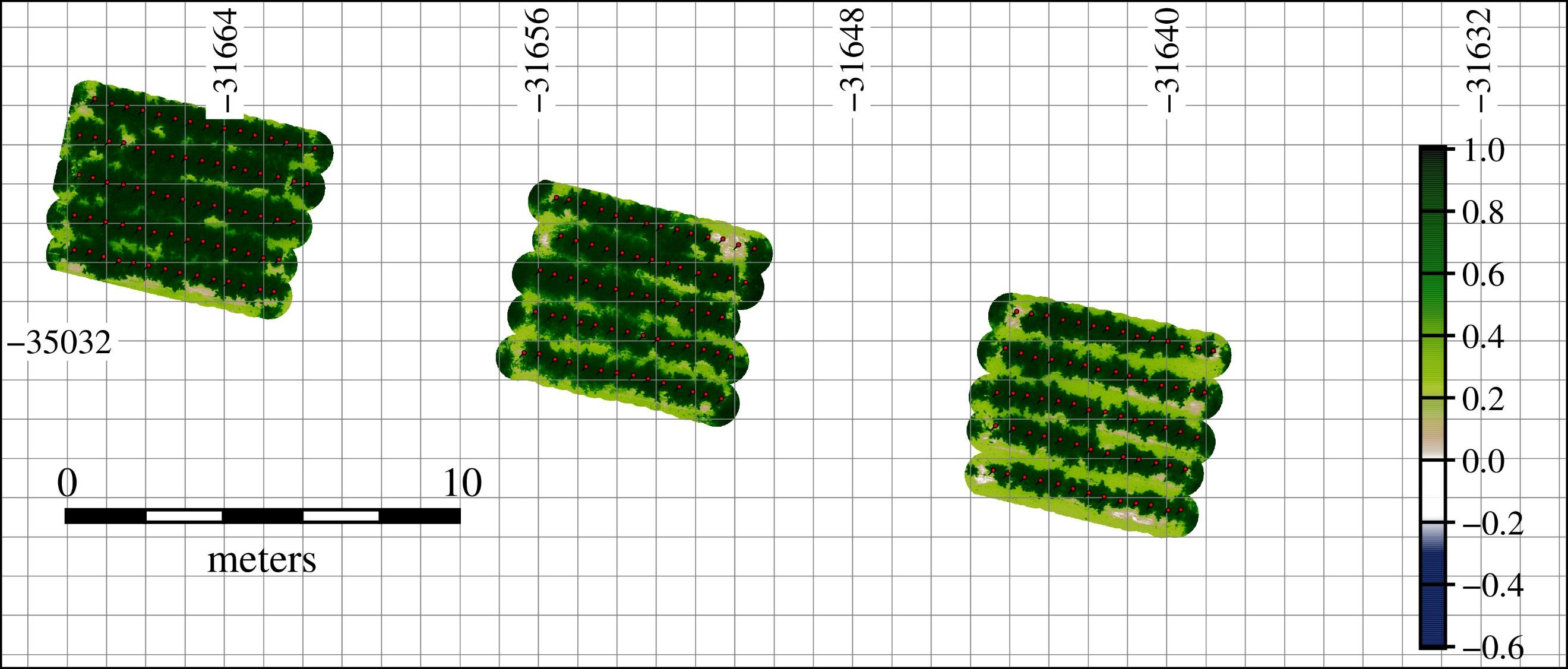  (f) |
| 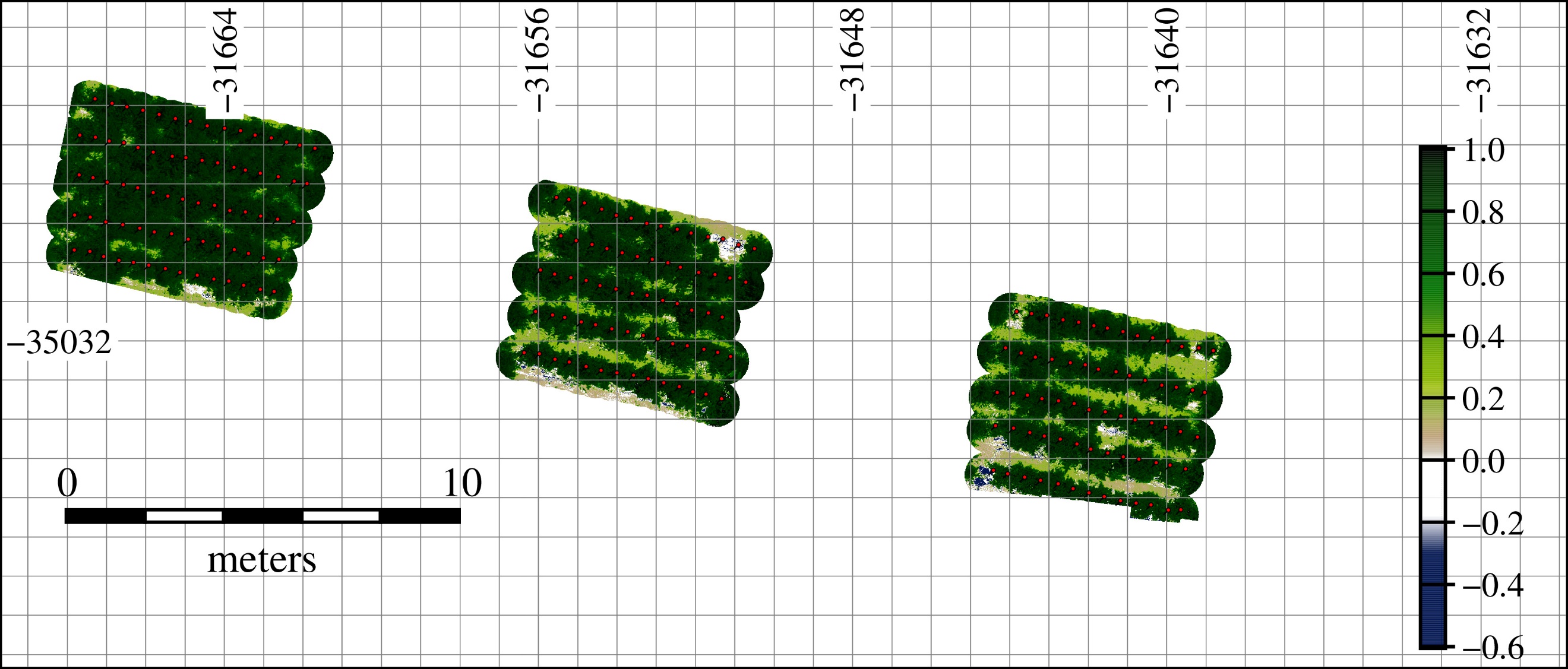  (g) | 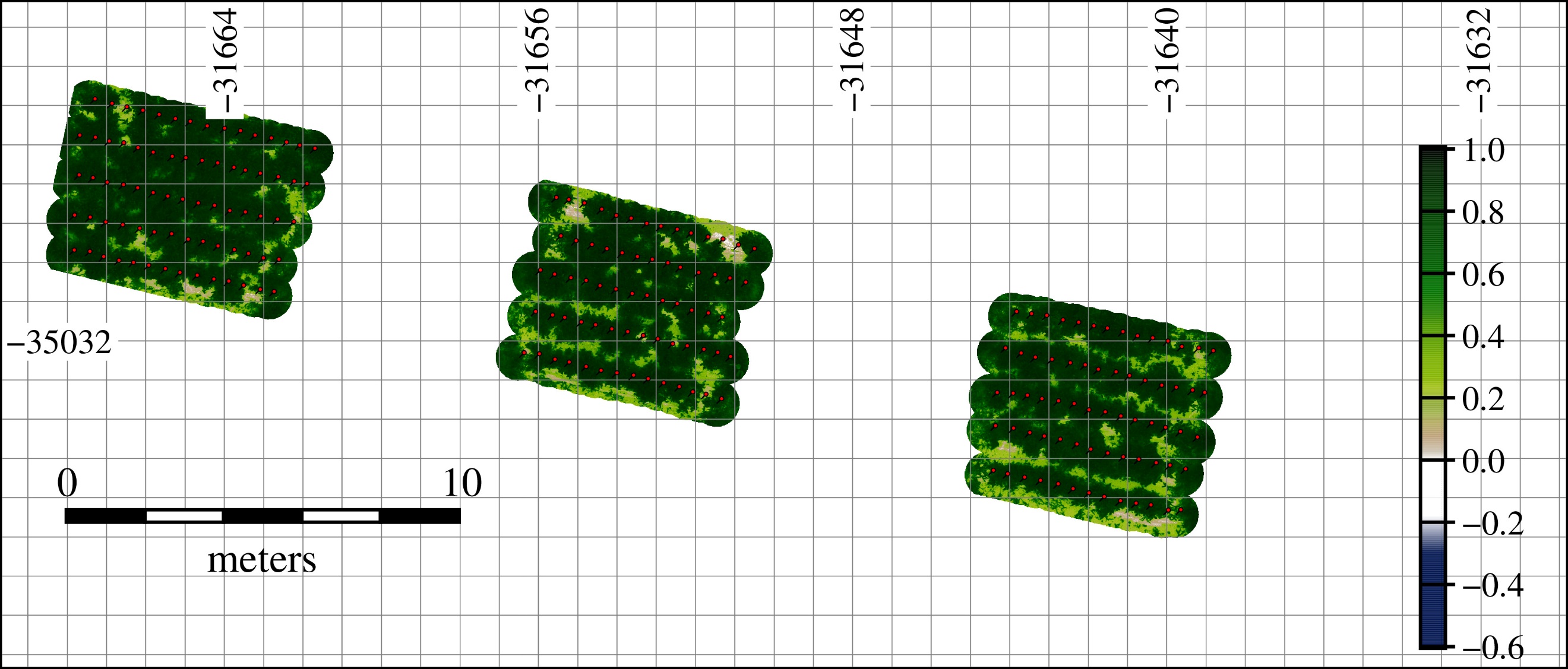  (h) |
| 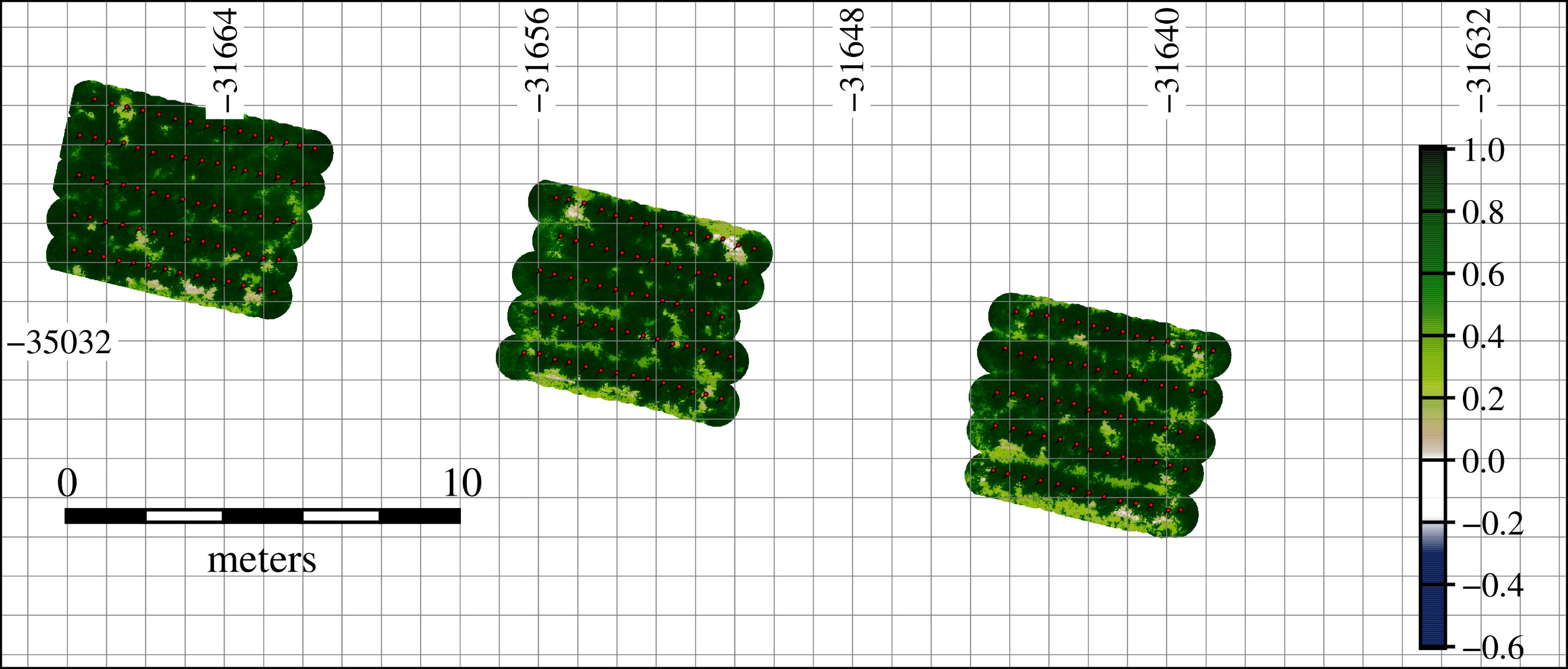  (i) | 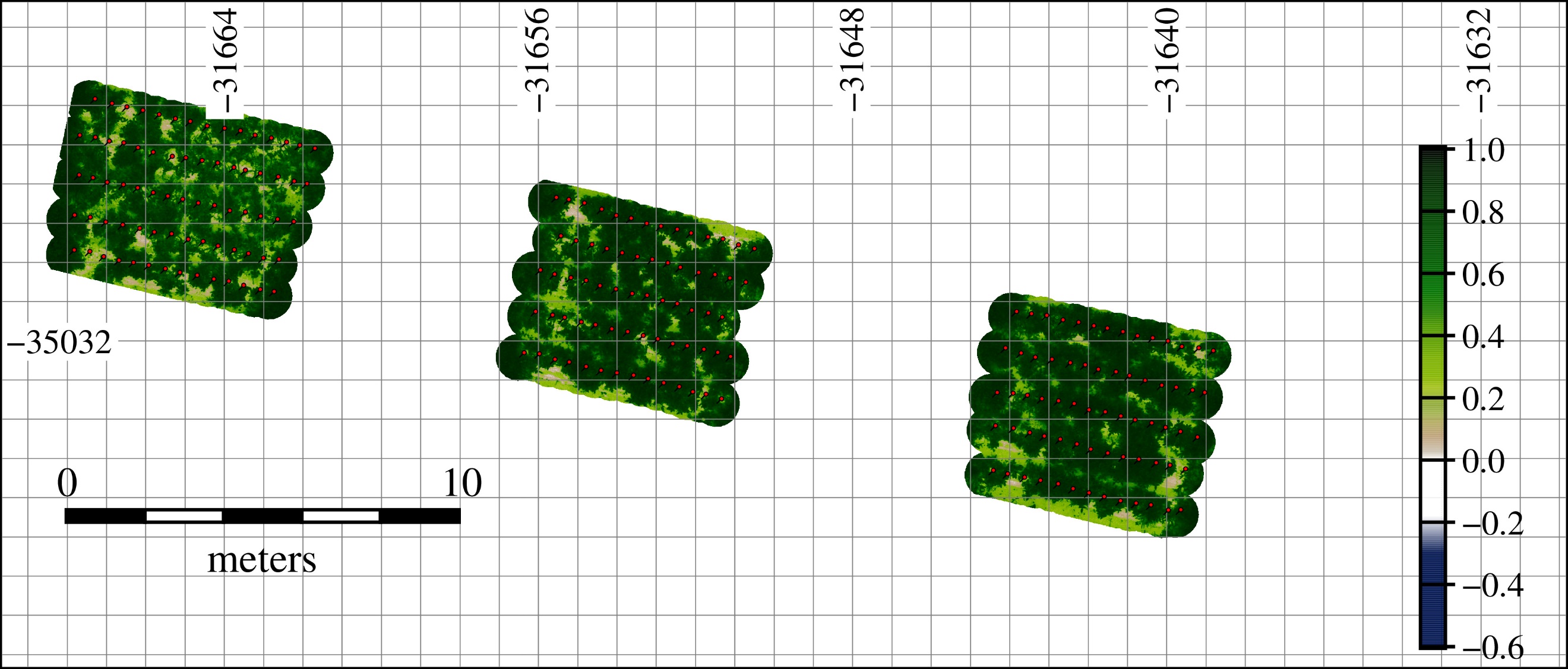  (j) |

**Fig. S1** Spatial multitemporal normalized difference vegetation index (NDVI) (–). **a** May 24, **b** May 30, **c** June 6, **d** June 11, **e** June 18, **f** June 26, **g** July 2, **h** July 12, **i** July 16, **j** July 24, 2020

| 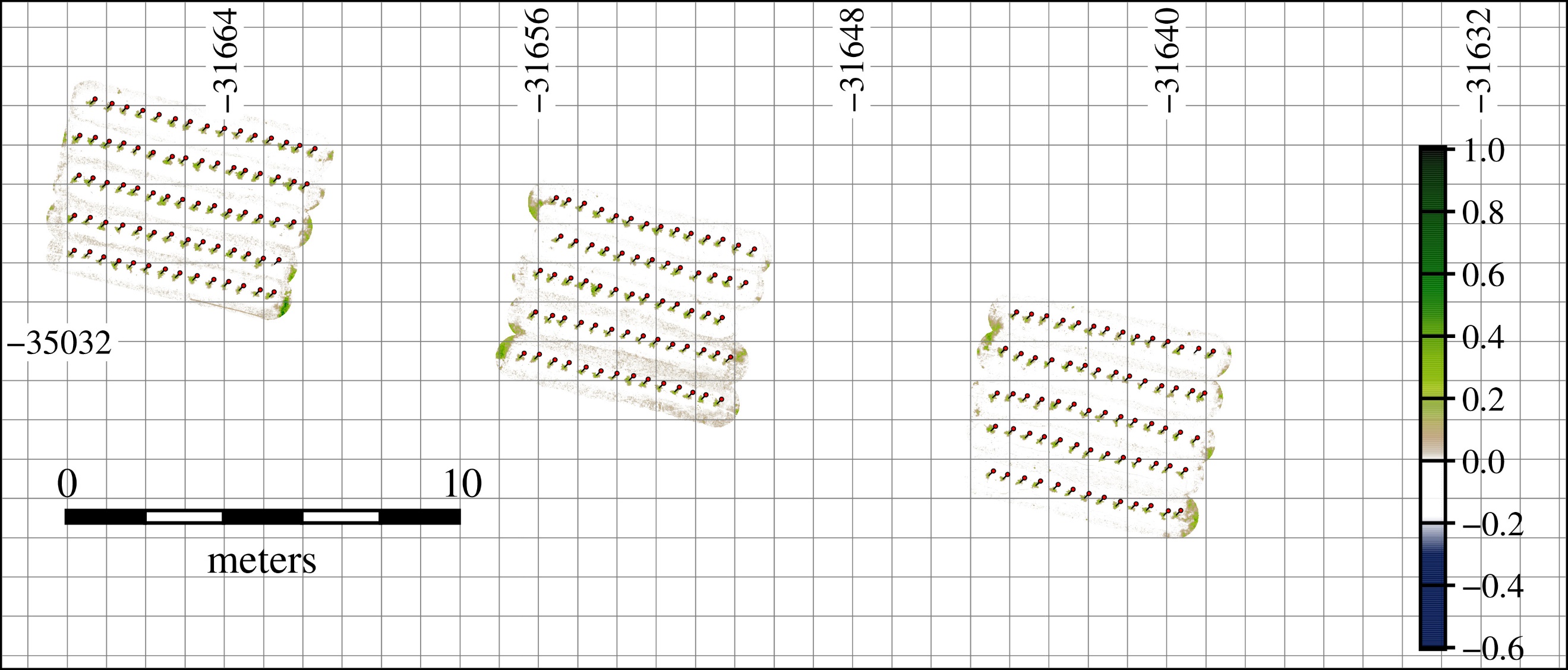  (a) | 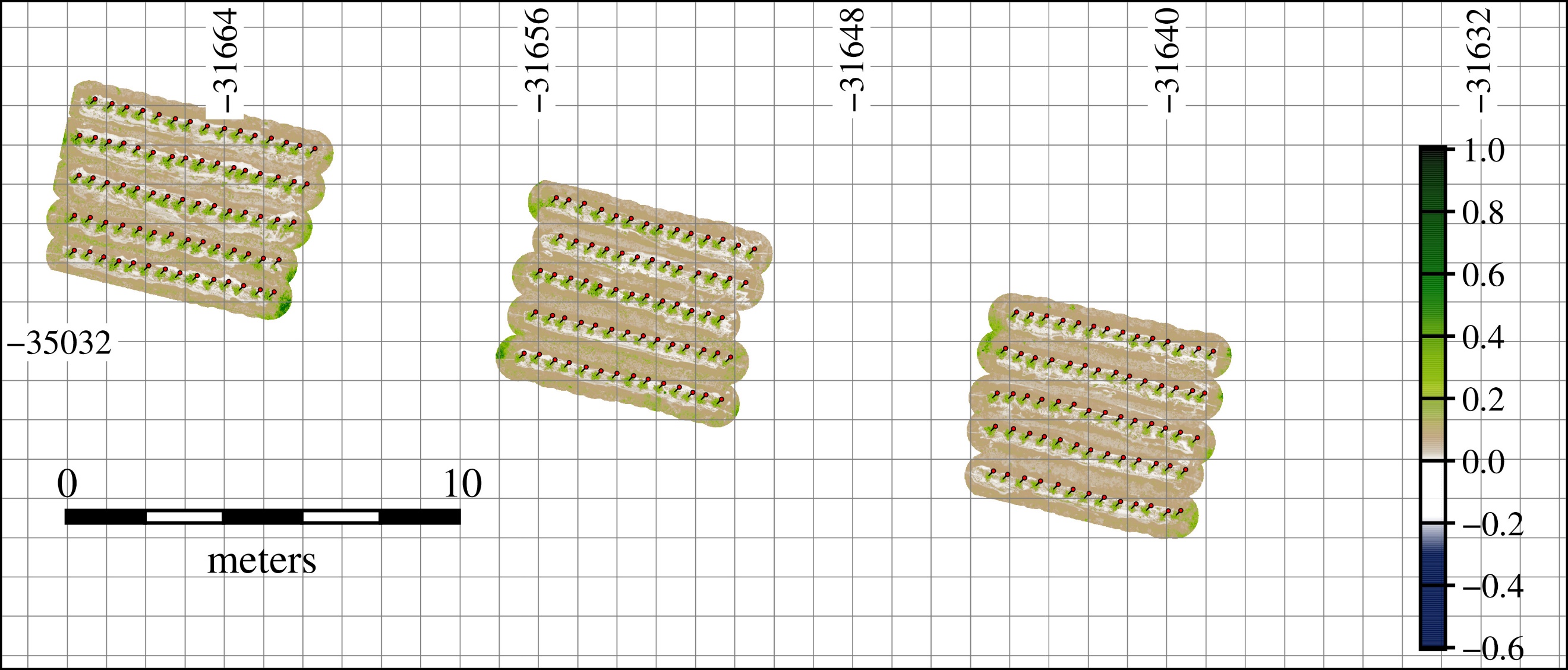  (b) |
| --- | --- |
| 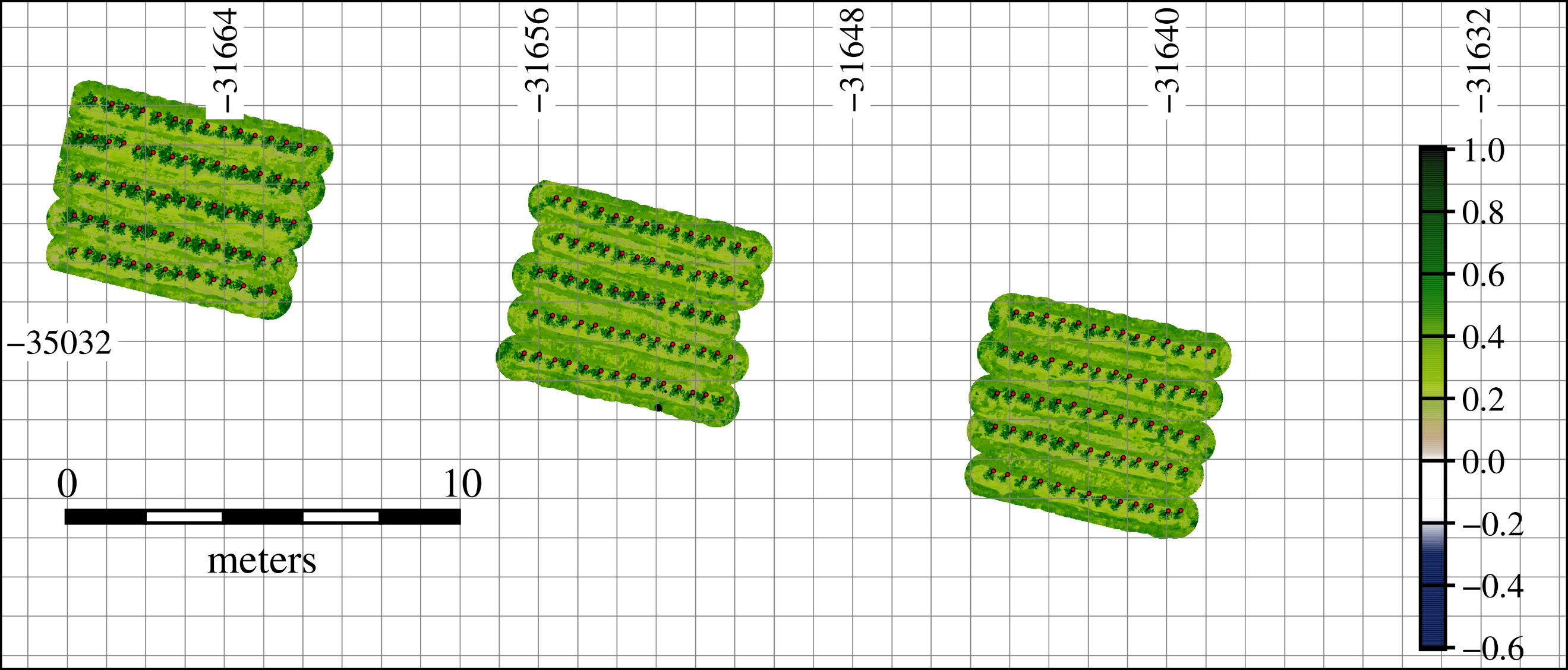  (c) | 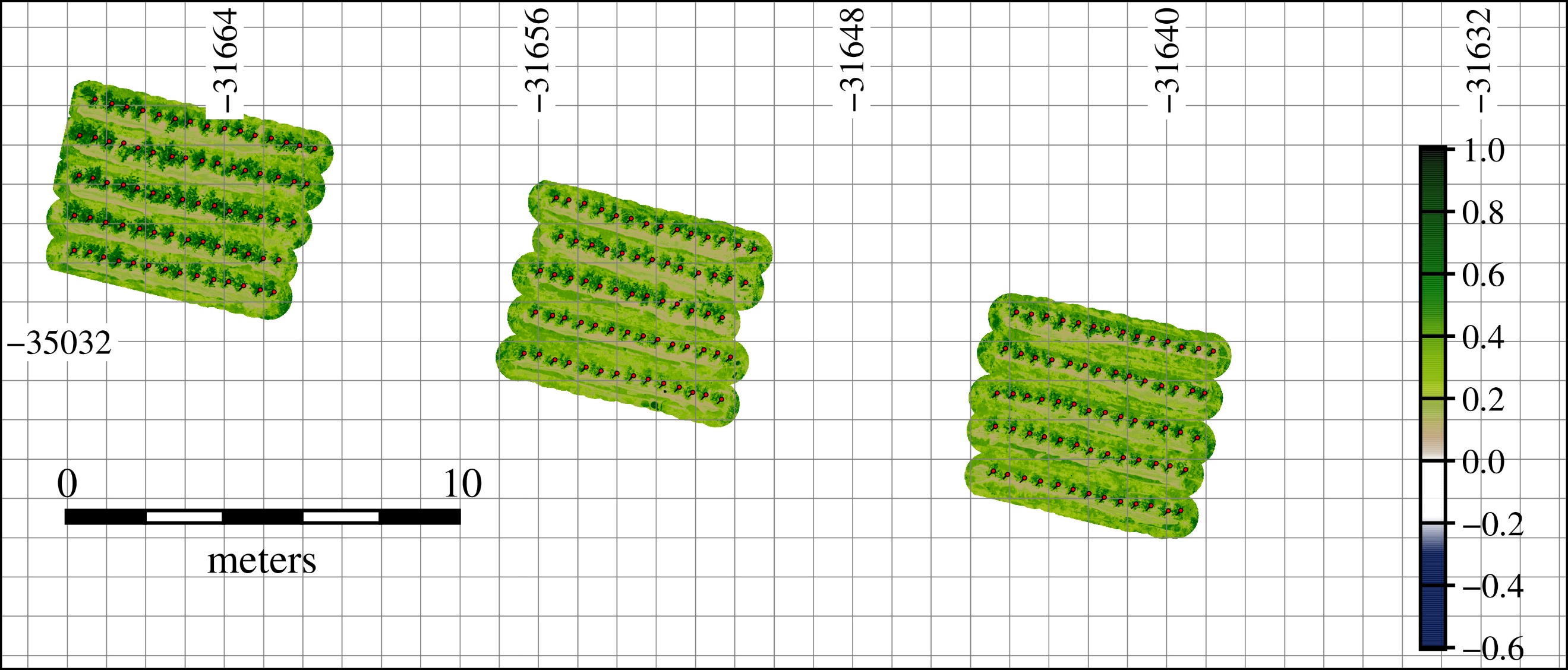  (d) |
| 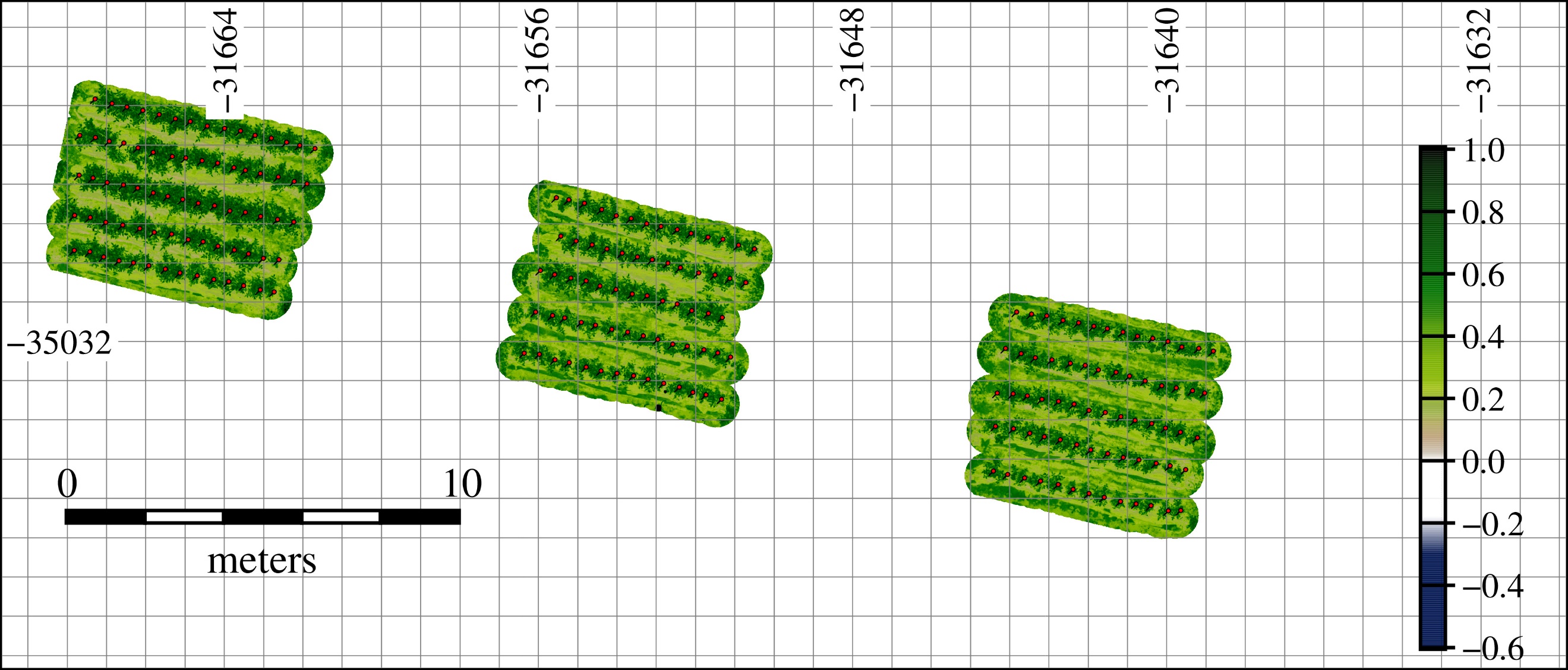  (e) | 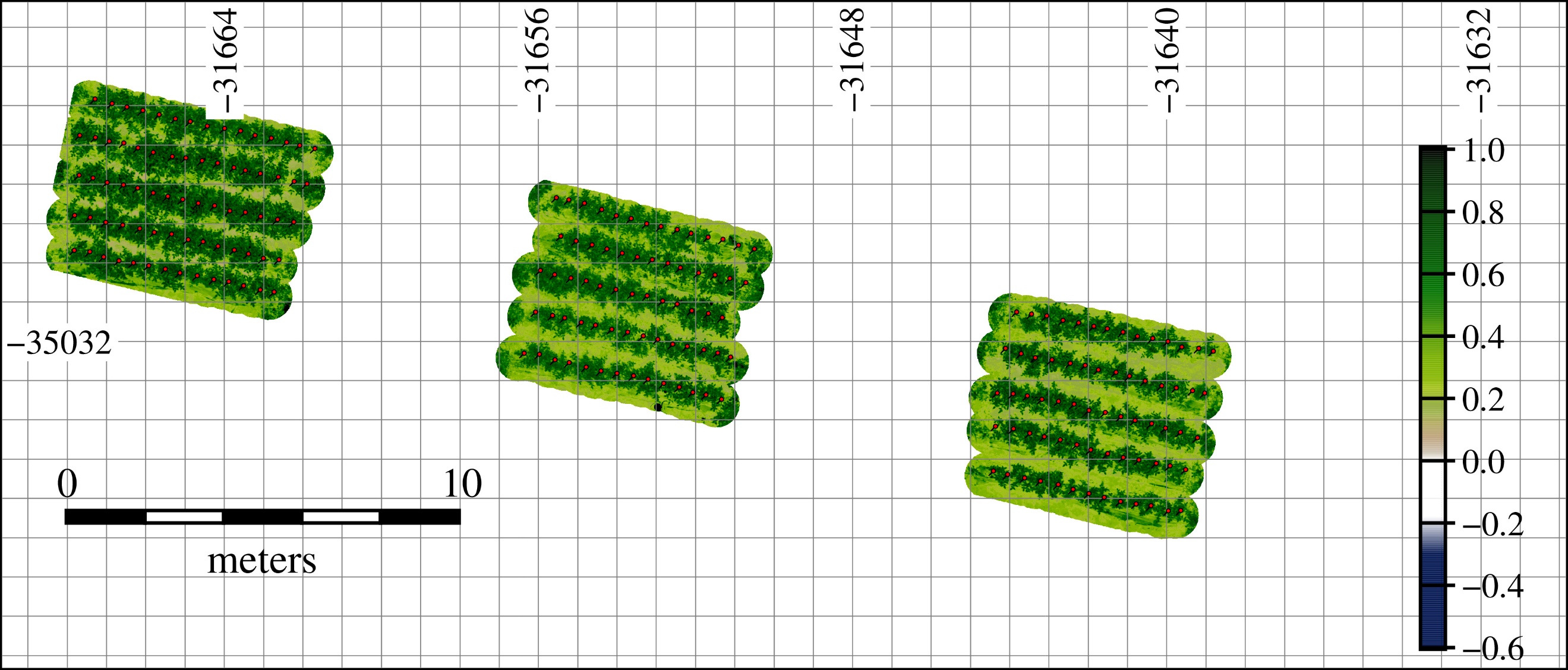  (f) |
| 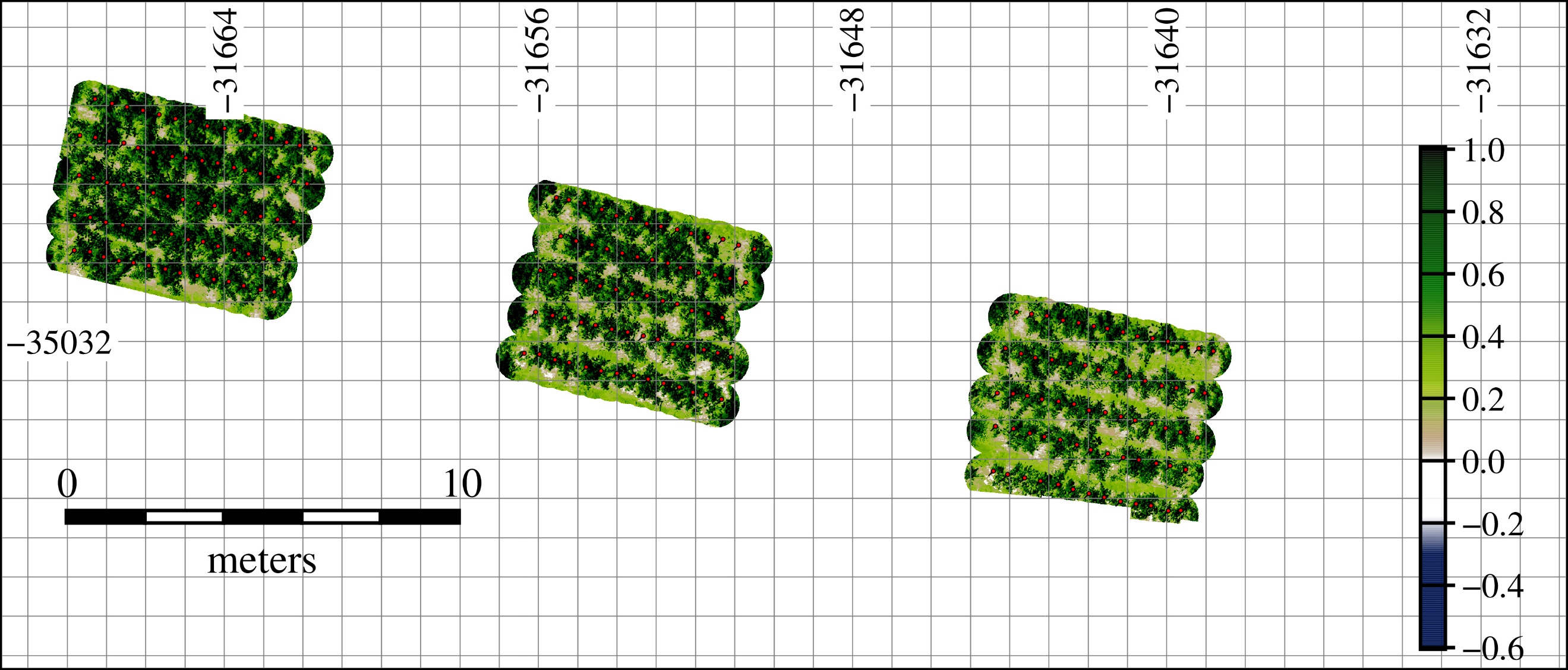  (g) | 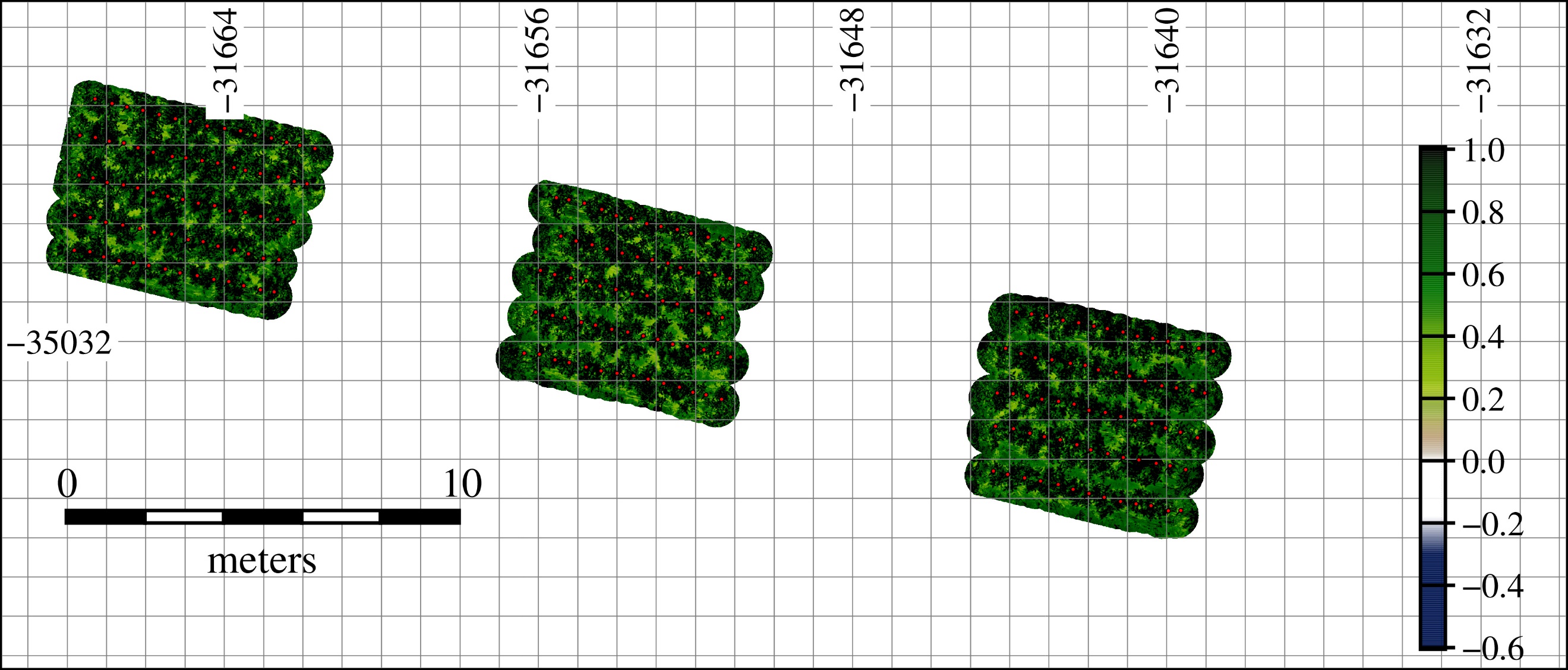  (h) |
| 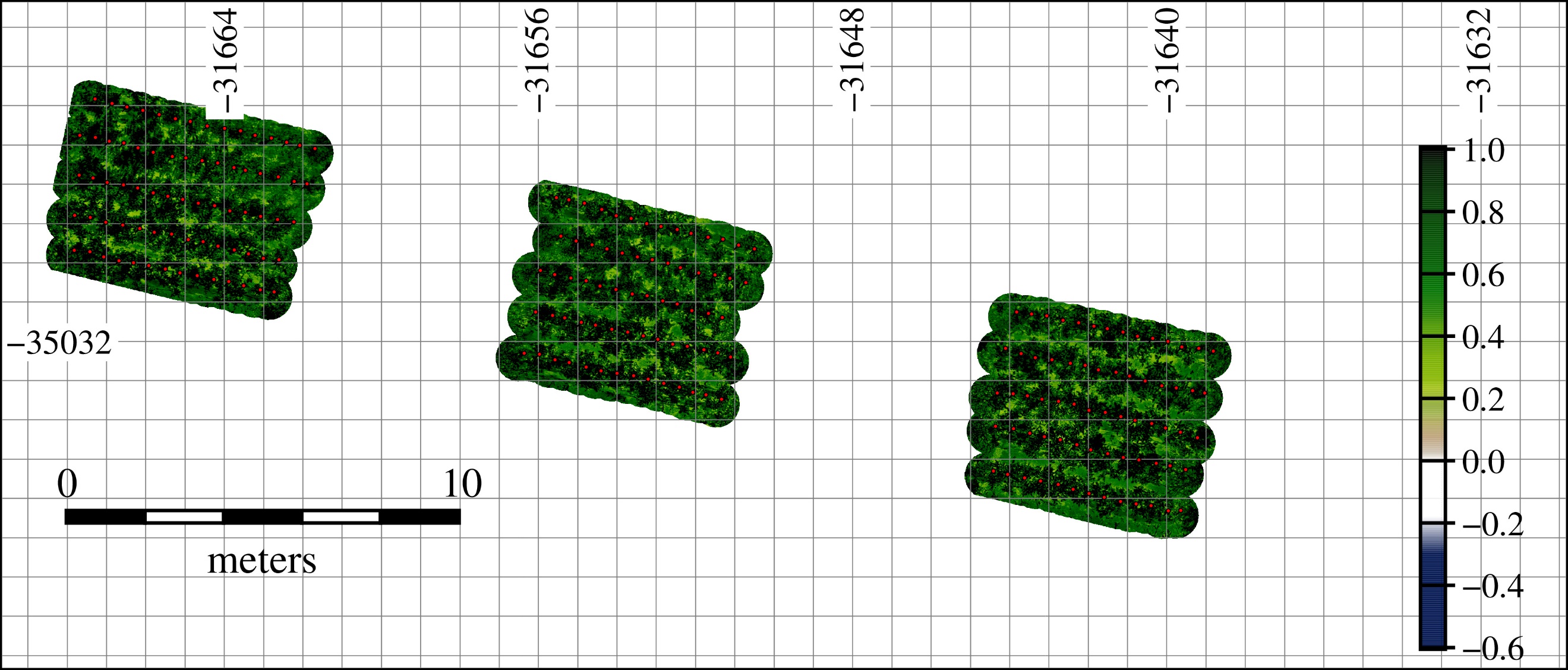  (i) | 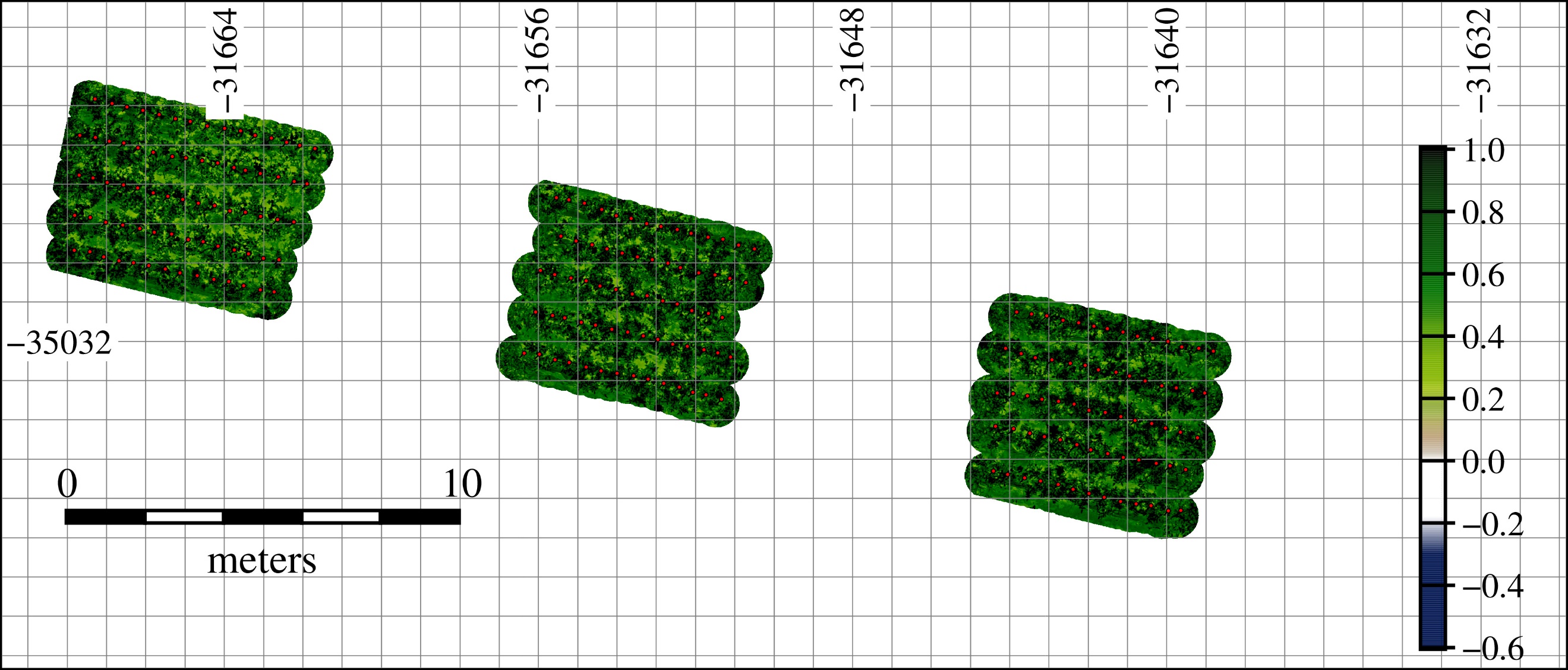  (j) |

**Fig. S2** Spatial multitemporal weighted difference vegetation index (WDVI) (–). **a** May 24, **b** May 30, **c** June 6, **d** June 11, **e** June 18, **f** June 26, **g** July 2, **h** July 12, **i** July 16, **j** July 24, 2020
